# Netter: re-ranking gene network inference predictions using structural network properties

**DOI:** 10.1186/s12859-016-0913-0

**Published:** 2016-02-09

**Authors:** Joeri Ruyssinck, Piet Demeester, Tom Dhaene, Yvan Saeys

**Affiliations:** Department of Information Technology, Ghent University - iMinds, IBCN research group iGent Technologiepark 15, Ghent, B-9052 Belgium; Data Mining and Modelling for Biomedicine group, VIB Inflammation Research Center, Ghent, Belgium; Bioinformatics Institute Ghent, Ghent University - VIB, Ghent, B-9000 Belgium; Department of Internal Medicine, Ghent University, Ghent, Belgium

**Keywords:** Gene regulatory networks, Network inference, Graphlets, Gene expression data

## Abstract

**Background:**

Many algorithms have been developed to infer the topology of gene regulatory networks from gene expression data. These methods typically produce a ranking of links between genes with associated confidence scores, after which a certain threshold is chosen to produce the inferred topology. However, the structural properties of the predicted network do not resemble those typical for a gene regulatory network, as most algorithms only take into account connections found in the data and do not include known graph properties in their inference process. This lowers the prediction accuracy of these methods, limiting their usability in practice.

**Results:**

We propose a post-processing algorithm which is applicable to any confidence ranking of regulatory interactions obtained from a network inference method which can use, *inter alia*, graphlets and several graph-invariant properties to re-rank the links into a more accurate prediction. To demonstrate the potential of our approach, we re-rank predictions of six different state-of-the-art algorithms using three simple network properties as optimization criteria and show that Netter can improve the predictions made on both artificially generated data as well as the DREAM4 and DREAM5 benchmarks. Additionally, the DREAM5 *E.coli.* community prediction inferred from real expression data is further improved. Furthermore, Netter compares favorably to other post-processing algorithms and is not restricted to correlation-like predictions. Lastly, we demonstrate that the performance increase is robust for a wide range of parameter settings. Netter is available at http://bioinformatics.intec.ugent.be.

**Conclusions:**

Network inference from high-throughput data is a long-standing challenge. In this work, we present Netter, which can further refine network predictions based on a set of user-defined graph properties. Netter is a flexible system which can be applied in unison with any method producing a ranking from omics data. It can be tailored to specific prior knowledge by expert users but can also be applied in general uses cases. Concluding, we believe that Netter is an interesting second step in the network inference process to further increase the quality of prediction.

**Electronic supplementary material:**

The online version of this article (doi:10.1186/s12859-016-0913-0) contains supplementary material, which is available to authorized users.

## Background

Network representations are widely used and vital in many areas of science and engineering. They serve both as an endpoint for users to structure, visualize and handle large amounts of data and as a starting point for various algorithms that use networks for automated hypothesis generation. In systems biology, one of the long-standing challenges is the reverse engineering of the cell’s transcriptome in the form of gene regulatory networks (GRNs). This has proven to be a daunting task, as the amount of genes in the network vastly exceeds the amount of available measurements. As a result, many computational methods have been developed [1–3] which try to overcome this challenge using various strategies. These methods differ not only in their accuracy to infer the network but also strike a different balance between scalability and complexity [4, 5]. In a recent community-wide effort, a large blind assessment of unsupervised inference methods using microarray gene expression data was conducted [6]. This study concluded that no single inference method performs best across all data sets but that in contrast, the integration of several techniques to form an ensemble ‘community’ prediction led to a robust and top performance. In a collaborative effort between the DREAM organizers, the GenePattern team [7] and individual contributors, a web service GP-DREAM was set up to run and combine current state-of-the-art methods. To date, five methods are offered: ANOVerence [8], CLR [9], GENIE3 [10], the Inferelator [11] and TIGRESS [12].

Common inference strategies of GRN inference algorithms include the calculation of local pairwise measures between genes or the transformation of the problem into independent regression subproblems to derive connections between genes. It is clear that using these schemes, the algorithm is unaware that the goal is to infer an actual network topology. Therefore, the global network structure cannot influence the inference process. For example, relevance networks [13] are created by calculating the mutual information between each possible gene interaction pair. A high mutual information score between a gene pair is then considered as putative evidence of a regulatory interaction. It is well known that this technique predicts a large amount of false positive interactions due to indirect effects. Two widely-used methods, CLR [9] and ARACNE [14] acknowledge this weakness and implement strategies to mitigate this problem by incorporating a more global network context. ARACNE uses the Data Processing Inequality on every triplet of genes to filter out the weakest connection. CLR builds a background model for each pair of interacting genes and will transform the mutual information score to its likelihood within the network context. WGCNA [15] also incorporates a global network context in the network reconstruction step of the algorithm. Pairwise correlations are raised to the optimal power to maximally fit a scale-free topology property of the constructed network. In a more general context, Network Deconvolution [16] was proposed as a post-processing technique to infer direct effects from an observed correlation matrix containing both direct and indirect effects. Similarly, a post-processing method named Silencer [17] uses a matrix transformation to turn the correlation matrix into a highly discriminative ’silenced’ matrix, which enhances only the terms associated with direct causal links. However, in general and as to date, almost none of the state-of-the-art algorithms make use of general or specific structural knowledge of gene regulatory networks to guide the inference progress. In contrast, such structural properties of GRN and biological networks in general have been studied extensively in literature [18, 19], introducing concepts such as modularity, hub-nodes and scale-free biological networks. The topology of a specific GRN is highly dependent on the experimental conditions and the type of cell [20] although general topological properties have been reported. It has been described that both prokaryotic and eukaryotic transcription networks exhibit an approximately scale-free out-degree distribution, while the in-degree distribution follows a restricted exponential function [21]. Furthermore, the concept of relatively isolated sets of co-expressed genes under specific conditions, called modules, has been introduced, as discussed in [22]. Topological analyses of GRN have also revealed the existence of network motifs [23], recurrent subgraphs in the larger network which appear more frequent than would be expected in randomized networks. The existence of such network motifs and their frequency of occurrence inevitably has an impact on the global network structure. Finally, prior knowledge about the topology of the specific GRN of the cell at hand can be available, in the simplest scenario in the form of already known regulatory links extracted from literature. We believe that the current non-inclusion of such known structural properties in the inference process leads to predictions that do not achieve their full potential. Furthermore, they are often characterized by different graph-invariant measures than networks found in literature. Although it is hard to completely transform these predictions into more realistic networks, it is clear that the inclusion of structural knowledge is desirable and will be beneficial to the prediction accuracy. However, including such complex and diverse topological information directly in the inference process of existing algorithms is non-trivial and offers little room for modifiability.

Instead in this work, we propose and validate a post-processing approach that aims to be easily modifiable and extendable. The resulting algorithm, named Netter, uses as input any ranking of regulatory links sorted by decreasing confidence obtained by a network inference algorithm of choice. It then re-ranks the links based on graph-invariant properties, effectively penalizing regulatory links which are less likely to be true in the inferred network structure and boosting others. It is not the goal of this work to put forth structural properties of GRN, instead we wish to offer a flexible system in which the end user can decide which structural properties are to be included or emphasized. Expert users can easily design and include novel structural properties and consequently tune Netter to a specific use case. However, to validate our approach, we also introduce and motivate three simple structural properties and default settings which can be used in a more general setting in which specific knowledge of the GRN is unavailable. Using these proposed structural properties and settings, we demonstrate that Netter improves the predictions of six state-of-the-art inference methods on a wide array of synthetic and real gene expression datasets including the DREAM4 and DREAM5 benchmarks. Netter also slightly improves the DREAM5 community prediction of the *E.coli.* network inferred from real expression data. We compare and discuss the performance of Netter with other techniques that aim to incorporate the global network or post-process GRN predictions. Next, we further investigate and discuss the characteristics of improvement of Netter. Lastly, we show that the performance gain of Netter is robust with regard to its parameter settings and the exact definition of its structural properties.

## Methods

**Fig. 1:**
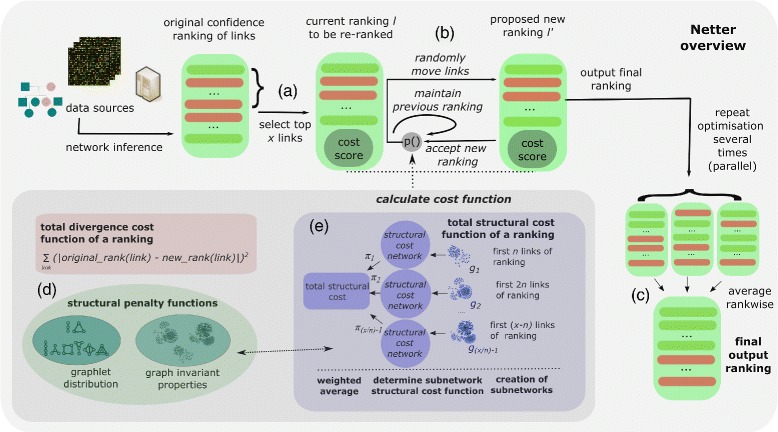
Overview of the re-ranking approach of Netter. A ranking of regulatory links sorted by decreasing confidence is assumed. This prediction can be obtained by an inference method of choice using any data source. In a first step, the top *x* links of the ranking are extracted. Netter will assign these links a new position, whereas the other links maintain their original ranks. The extracted ranking is awarded a cost and using simulated annealing the cost function is minimized several times, obtaining re-ranked lists in the progress which are then averaged to obtain the final output ranking. The cost function strikes a balance between modifying the original ranking to have better structural properties while remaining true to the original ranking

Figure 1 shows an overview of the Netter algorithm. In the following subsections, we first formalize the problem statement and elaborate on the different steps of the algorithm. Next, we introduce three different structural properties which will be used to re-rank the input networks. Finally, we discuss the different network inference methods that will be used to create the input networks and briefly discuss the computational aspects of Netter. In the process of formalizing the problem, we will introduce a parameter for every design decision. In practice however, many of these parameters do not substantially influence the outcome of Netter and do not need tuning as we will further discuss in the Results and discussion Section.

### Input, problem definition and output

Most GRN inference methods return an absolute ranking of all possible edges, sorted by decreasing confidence that this link is a true regulatory interaction. This ranking is then later transformed into a network representation by selecting a threshold determined by the algorithm or end-user. Netter uses as input such an absolute ranking of potential gene regulatory links. This ranking can be incomplete, however no regulatory links will be added as Netter is strictly a re-ranking approach. No further assumptions are made about which algorithms or data sources were used. Although we focus here on unsupervised network inference methods which use microarray expression data to infer network topologies, Netter is generally applicable to any method producing a ranking from omics data. In practice, it only makes sense to re-rank the top of the ranking noting that networks consisting of 100 genes already produce a complete ranking of 9900 potential regulatory links (excluding self-regulatory interactions). Therefore, in the first step of the algorithm (Fig. 1a), the top *x* most confident links of the prediction are extracted, where *x* is a user-chosen value. The algorithm will work on these links only, assigning them a new rank, whereas the remaining links maintain their original ranks and cannot influence the decision process.

### Formulation as an optimization problem

Using the extracted top *x* links, an optimization procedure is started which is performed several times and can be executed in parallel (Fig. 1b). Each optimization procedure outputs a new ranking, after which the final ranking of Netter is obtained by averaging rank-wise over all output rankings (Fig. 1c). Averaging over multiple output rankings is a crucial step in Netter. It guarantees robustness and performance gain as the total cost function which is optimized is non-convex with many local optima. We will further discuss this in the Results and discussion section. A single optimization procedure tries to find a ranking *l*∈*L*, the set of all possible rankings, which minimizes a total cost function *f* assigning a positive value to any ranking in *L*.

$${\min_{l \in L} f(l)}, \text{with}\,\, f:L \rightarrow \mathbf{R}^{+} $$

This total cost function is defined as the weighted sum of two cost functions *s* and *Δ* with the same (co)domain as *f*:

$$f(l) = s(l) + \alpha. \Delta(l) $$

Here *α* is a global balance factor, *s* is a structural cost function giving a score to a ranking based on structural properties and *Δ* is a divergence function quantifying how much a ranking is different from the original ranking. Intuitively, *f* strikes a balance between modifying the original ranking to have better structural properties while remaining true to the original ranking (Fig. 1d).

### Simulated annealing optimization

This optimization problem is then solved by following a simulated annealing approach to the problem. In a single step of the optimization process, we create a new ranking *l*^′^ by randomly moving *γ* links up or down the ranking by *θ* positions. *γ* is sampled uniform from [ 1,*Γ*] in each step, while *θ* is sampled uniform for each link from [−*Θ*,+*Θ*]. *Θ*, *Γ* being user-set integer values. In practice, this way the optimization process will explore both minor as substantial changes to the ranking. The newly generated ranking *l*^′^ is accepted as the new ranking if *f*(*l*^′^)<*f*(*l*) or with a probability of $\phantom {\dot {i}\!}e^{-(\,f(l^{\prime })-f(l))/T}$ otherwise, with *T* being the current temperature of the annealing process, as proposed by [24]. We use a traditional exponential cooling schedule in which the temperature is repeatedly lowered by a constant factor *μ* after each iteration. To avoid manual tuning of the annealing parameters for each network, Netter will automatically adjust the parameters and restart if the acceptance ratio of bad mutations during a starting window (e.g. the first 10 % iterations) is not within user-defined limits.

### Assigning a structural cost function and a divergence cost function to a ranking

In Netter, the structural cost function *s* assigns a score to a ranking *l* based on topological properties. We adopt the following procedure (Fig. 1e) to transform a ranking into network representations of which structural properties can be calculated. In a first step, a subnetwork *g*_1_, containing *n* links, is created by using the top *n* links of the ranking. Next, a subnetwork *g*_2_ is created, containing the first 2*n* links of the ranking. This process is repeated until a subnetwork *g*_[(*x*/*n*)−1]_ is created, containing all but the last *n* links of the ranking. Summarizing, subnetworks *g*_1_, *g*_2_, …, *g*_*i*_, *g*_[(*x*/*n*)−1]_ of increasing size are created from the ranking *l*, consisting of *n*, 2*n*, …, *i*.*n*, *x*-*n* links respectively. We can then calculate a score for each of these subnetworks by using a structural property function *s*_*struct*_ which depends on some structural properties. *s* is then defined as the weighted sum of the structural scores of the individual subnetworks *g*_*i*_ created from the ranking *l*.

$$s(l) = \sum\limits_{i} \pi_{i}. s_{struct}(g_{i}) $$

The coefficients *π*_*i*_, each associated with a subnetwork, are set to decrease according to the network size. Smaller subnetworks, corresponding to the top of the ranking are in this way more influential in the total structural cost of the ranking. Intuitively, this way the optimization procedure will make the top of the ranking correspond more to structurally realistic networks by moving links to the top of the ranking which structurally improve the network and move down others which seem odd to be present. As the size of the search space of possible rankings allows for an almost infinite amount of rankings which effectively minimize the structural cost function close to its lowest possible value, the divergence function *Δ* needs to be included in the total cost function *f*. This function thus acts as a regularization term and is defined as:

$$\Delta(l) = \sum\limits_{link} ({|original\_rank(link) - new\_rank(link)|}^{2}) $$

#### Structural property functions

The structural property function *s* is defined as the weighted sum of individual structural penalty functions *s*_*struct*_ which each have a user defined weighting coefficient. The amount of structural properties which one could associate with a typical GRN are plenty and are much subject to debate. Furthermore, some structural properties are highly dependent on the cell at hand or the experimental conditions. Therefore, Netter is designed to allow for the easy inclusion and exclusion of new or existing structural properties. Expert users or researchers which have prior knowledge can tune Netter to specific use cases. For example, a custom penalty could be defined which penalizes the non-inclusion of known interactions. It is not the main focus of this work to develop or suggest (complex) structural penalty functions. However, to validate our re-ranking approach, we introduce several general structural properties based on graph-invariant properties and graphlets. In this article, we restrict these functions to be a simple v-shaped mapping of a certain structural property of the network *y* to a cost value, although Netter can include any function that maps a network to a positive value. The v-shaped function is defined as follows:

$$s_{struct}(\,y) := \| ay +b \| $$

Here, the parameters *a* and *b* can be specific for each of the *s*_*struct*_ and the default values can be found in Additional files 1–3. In the results section we discuss how changes in the relative weighing coefficients and the exact shape of the individual structural penalty functions (by varying *a* and *b*) influence the performance of Netter.

#### Graphlet-based structural penalty

**Fig. 2:**
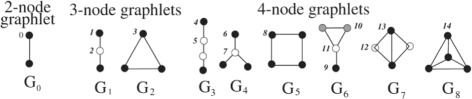
All 3-node and 4-node connected network graphlets. Figure adapted from [26]

Graphlets have been introduced as small connected non-isomorphic induced subgraphs of a larger network [25]. They differ from the concept of network motifs by the fact that an induced subgraph needs to contain all the edges between its nodes which are present in the parent network. Since the original study, several other graphlet-based network properties have been derived and successfully applied in various applications [26]. If we focus on 4-node graphlets, it is clear that hub-like structures in the network will promote the occurrence of G4 (see Fig. 2) graphlets. We postulate that the relative frequency of G4 graphlets as compared to all other 4-node graphlets could be used as an optimization criterion to create networks which are more modular. The need for increased modularity can be motivated by the fact that in the inferred networks, the network topology resembles a full-mesh structure as opposed to a scale-free, modular topology that is generally associated with a GRN. To be precise, we created a graphlet-based penalty function which defines *y* as the relative percentage of G4 graphlets compared to all other 4-node graphlets. Next, *a* and *b* are set in the v-shaped cost function such that a lower cost corresponds to networks with a high relative percentage of G4 graphlets. Including this penalty does not eliminate the possibility of other valid biological structures to appear in the network (e.g. feed forward loops), as strong signals will always be retained due to the divergence cost penalty or other included penalties. This penalty will merely discourage the occurence of weak signals connected to stronger signals (links that appear at the top of the original ranking) that would result in less modular networks. In practice, penalties based on other graphlets can (and are encouraged) to be included in Netter to further refine the desired network shape. One can also include penalties based on subgraph counts in a directed network context (i.e. network motifs). However, for demonstration purposes, we will only include the G4-graphlet relative frequency as optimization criterion as we believe it is the most simple and intuitive criteria. In the Results section and in Additional file 1 we discuss the default mapping (*a* and *b*) and the stability of this penalty.

#### Regulatory gene limiting penalty

In the case a list of known regulatory genes is not available, as in the DREAM4 benchmark, predictions tend to favor the presence of outgoing links originating from almost every node in the inferred network. This is due to indirect effects, if for example a gene A regulates both genes B and C in the same process, most algorithms will also predict a regulatory link between B and C. Furthermore, in the absence of interventional or time-series data, the direction of a regulatory link is hard to predict resulting in a large amount of bi-directional links as both directed edges will usually be close to each other in the ranking. In reality, it is improbable that every gene in the network has an outgoing link in the network, as this would suggest that the gene has a regulatory function. Although the graphlet-based structural penalty partially addresses these problems, a simple regulatory gene limiting penalty was created which defines *y* as the amount of nodes in the network with at least one outgoing link relative to the total amount of genes in the network. Parameters *a* and *b* were set such that a high cost was associated with networks containing a high percentual number of nodes that have outgoing links. Additional file 2 describes the exact default mapping and a more detailed performance stability analysis.

#### Anti-dominating penalty

In some cases after re-ranking, we noticed that a regulatory gene and its target genes would completely dominate the top of the prediction list, leaving no room for other modules. This behavior is unwanted, as one wants to discover different areas of the network. This penalty counters this problem by penalizing a percentual large amount of links originating from the same gene in the network. The anti-dominating penalty defines *y* as the ratio between the maximum amount of links originating from a same gene in the network and the total amount of links in the network. Additional file 3 describes the default mapping and a stability analysis of this penalty.

### Computational aspects of Netter

The large search space of possible rankings results in the necessity of performing many steps to minimize the optimization function. Therefore, it is critical that a single step is performed as efficient as possible. Two computationally expensive processes can be distinguished in a single iteration. First, the new candidate ranking *l*^′^ created from *l*, needs to be transformed into new subnetworks *g*_*i*_. Second, structural penalties need to be calculated using the newly created subnetworks of which some, e.g. the graphlet count, can be computationally expensive. Executing both processes from scratch would result in an unacceptable runtime. However, because the new ranking *l*^′^ is similar to the current ranking *l* an incremental approach to the problem can be used. Therefore, Netter uses an incremental update scheme to keep track of the subnetworks and can efficiently revert back in case the new ranking is rejected. All penalty functions, including the graphlet enumerator have been defined and developed to work in an incremental way and new structural penalties should also be implemented in this setting. Each optimization procedure in Netter is ’embarrassingly parallel’. Therefore, Netter will assign new optimization runs to each idle, available core. To give an estimate of the execution time of Netter: a typical set-up as described further including 100 independent optimization runs, took 5 single core hours on a Intel i3 CPU M350 clocked at 2.27 GHz, 8.00 GB of RAM and a 64-bit OS. However, the running time is highly dependent on the parameter settings and the list of included penalties. Furthermore, the amount of independent runs (=100) is conservative and can be further lowered if computing power is an issue. We discuss this in more detail in the Results and discussion subsection.

### Selected network inference methods

In order to test Netter we performed a large number of experiments using a variety of network inference methods. We selected six network inference methods in total with varying complexity and performance. In addition, in case of the DREAM5 networks, the community prediction networks as created and published in [27] were added. Of the six selected network inference methods, three are based on mutual information scores: CLR [9], ARACNE [14] and BC3NET [28]. Three other methods use machine learning techniques to infer the network GENIE3 [10], NIMEFI [29] and TIGRESS [12].

### Selected data sets and evaluation measures

Netter’s performance was evaluated using the five expression datasets from the DREAM4 *in silico* 100 multifactorial [27, 30, 31] challenge and the two expression compendia from the DREAM5 network inference challenge [6]. Furthermore, to avoid overfitting specific structural properties of these benchmarks, we created an additional 25 networks of different dimensions and associated expression compendia using two different synthetic gene expression data generators SynTRen [32] and GeneNetWeaver [30, 33]. Table 1 provides an overview of the dimensions and properties of the datasets. Using all of these datasets, we inferred the network topology using the algorithms described in the next subsection. Next, we chose a cutoff value *x* and re-ranked the resulting prediction using Netter. As evaluation measure, we consider both the Area Under the Receiver Operating Characteristic curve (AUROC) and the Area under the Precision-Recall (AUPR) curve, only taking into account the true edges present in the first *x* predicted links of the original ranking. Gold edges which are not present in this original ranking are removed from the gold standard prior to calculating the scores. This allows for a fair comparison between the original ranking and the re-ranked list as Netter is strictly a re-ranking algorithm and cannot add any edges outside the selected *x* edges. Furthermore, it allows a more clear comparison between networks of different dimensions. As a result, the AUROC and AUPR scores in this article depend on the original predicted ranking and cannot be compared between different methods. For some of the additional tests, a reduced dataset of 15 networks was used instead of the full dataset to ease the computational demands. This networks in this dataset were randomly selected from the full dataset and contain only GENIE3 predictions. For each test, we will clearly indicate if the full or reduced dataset was used.

**Table 1:** Overview of the datasets used in the performance tests

Name	Networks	Reg.genes	Genes	Samples	Type
DREAM4	5	100	100	100	Artif.
DREAM5 artif.	1	195	1643	805	Artif.
DREAM5 *E. coli.*	1	334	4511	805	Real
SynTRreN-100	5	100	100	100	Artif.
SynTRreN-150	5	150	150	150	Artif.
GNW-200	15	200	200	200	Artif.

## Results and discussion

To interpret the performance results of Netter, it is important to note that from a theoretical point of view, a post-processing approach can never improve every network prediction it is applied on. If this would be the case, repeatedly applying this algorithm on the outcome of a previous re-ranking would eventually result in the perfect ranking. An analogy can be found in lossless compression, where one also tries to find general properties to obtain a good compression ratio for a large set of probable items sampled from the population. In the specific case of Netter, each consecutive re-ranking will result in less information of the original prediction being used to guide the re-ranking process and therefore should be avoided. Furthermore, for a specific network it is hard to explain why a loss in prediction accuracy occurred. A possible reason is that the initial prediction was of insufficient quality to guide to optimization process in the right direction. It is known that these network inference algorithms achieve low accuracy and that algorithms can produce different rankings even with those obtaining similar performance metric scores [6, 29]. Further on in this section, we will briefly discuss the performance gain of Netter with regard to the initial prediction accuracy. Also in the following subsections, we present the results of performance tests, compare Netter to other similar technique, discuss the effect of successive applications of Netter and comprehensively investigate the influence of the various parameters settings and choice of the structural cost function definitions.

### Performance tests

We ran Netter on all networks and all method predictions using the following settings. The cutoff value *x* was set to the first 750 links or the amount of non-zero links in the case less edges received a non-zero score. The mutation parameters *Θ* and *Γ* were set to 70 links and 50 positions respectively. The subnetwork size parameter *n* was set to 25 and the associated coefficients *π*_*i*_ were set to 0.5*i*, for *i*= [1…amount of subnetworks]. The annealing scheme allowed an acceptance ratio of bad mutations of approximately 10 % after the first 3000 of 30,000 iterations. The optimization process was performed 100 times for each prediction before averaging and all penalty functions discussed in the previous section were included. The relative weighing parameter was set to 25 for the graphlet penalty, 2 for the gene regulatory penalty and 75 for the anti-dominating penalty, *α* was set to 10^−5^. The influence of the individual penalty cost function shape, the relative weighing coefficients and other parameters on the performance is discussed in the next section. Each re-ranking experiment was repeated three times and, due to the ensemble approach of Netter, the rankings were almost identical.

**Fig. 3:**
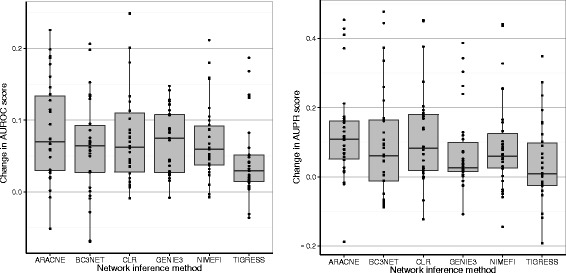
Change in AUROC and AUPR scores after applying Netter. Change in AUROC and AUPR scores after applying Netter on all datasets except DREAM-5 which are shown in Table 2. The different bars represent the network inference algorithm used to create the initial network. Each dot on the figure is a different re-ranked network and is the result of a single Netter re-ranking procedure consisting of 100 averaged independent optimization runs

Figure 3 shows the change in AUPR and AUROC compared to the original ranking after applying Netter on all datasets except DREAM5, each dot resembles a re-ranked network. For more details we refer to Additional file 4 which includes the evaluation metrics for each network. The results show that Netter substantially increases the prediction accuracy in the majority of cases across all algorithms. For the AUROC scores, the boxplot bars remain well above the baseline of 0.0, with only a few re-rankings resulting in a decrease in performance. Looking at the AUPR, the increase in performance is more pronounced compared to the AUROC change, with some re-rankings achieving an increase in AUPR of more than 0.25, which in some cases nets to a relative increase of more than 100 % compared to the original ranking.

**Table 2:** AUPR before and after re-ranking predictions of the DREAM5 dataset

Net.	GENIE3		NIMEFI		TIGRESS		Community	
	Orig.	New	Orig.	New	Orig.	New	Orig.	New
Artif.	0.94	0.96	0.81	0.82	0.92	0.90	0.91	0.88
E. coli.	0.15	0.21	0.18	0.21	0.20	0.16	0.13	0.15

**Fig. 4:**
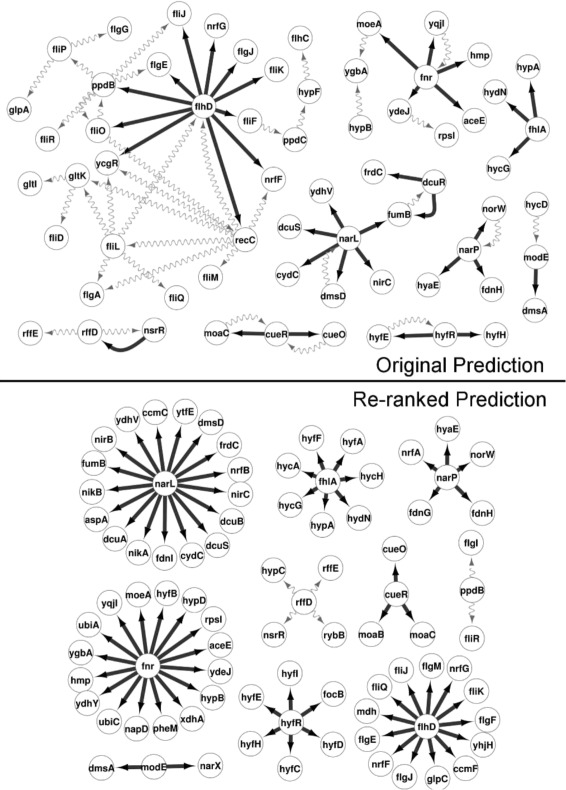
Network comparison view of a GENIE3 prediction before and after the re-ranking procedure of Netter. The first 75 links of each ranking are plotted. True positive links are shown as black solid lines, whereas grey curved lines indicated false positives

**Fig. 5:**
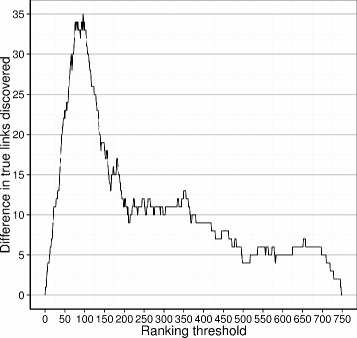
The difference in the amount of true links discovered at various thresholds for a re-ranking. At every possible threshold of the ranking, the amount of true positive links discovered by the original ranking is subtracted from the amount of true positive links discovered by the re-ranked network. The network is the same as the one plotted in Fig. 4

To give a more intuitive view on the accuracy gain, we take a closer look at a network (GNW-network 2) on which a substantial improvement was achieved. Figure 4 shows a network comparison view between the original GENIE3 ranking and the re-ranked list in which the first 75 links are plotted. The true positive links are shown as black solid lines, whereas grey curved lines indicate false positives. The resulting networks have 31 out of 75 of their predicted links in common. In the original, there were 36 true positive links, while the re-ranked prediction contains 69. Of the 36 true positives in the original prediction, 28 are still in the re-ranked network while 41 of the 44 new links entering the network are true positives. Further analysis shows that especially the top of the ranking is improved (Fig. 5). Indeed, for this example the first false positive is encountered at position 50 for the re-ranked list and at position 1 for the original. The fact that the improvement occurs at the top of the ranking is a desirable feature in practice.

Focusing on DREAM5, Table 2 shows an overview of the AUPR of GENIE3, NIMEFI, TIGRESS and the community network. We did not re-rank the predictions of the mutual information methods, as these methods were outperformed by the former in the DREAM5 challenge. The table shows that the original AUPR score on the artificial network is already quite high and Netter is unable to further improve the prediction. However, on the *E. coli.* network inferred using real expression data, Netter substantially improves the predictions of GENIE3 and NIMEFI while the TIGRESS performance decreases. Netter is also able to slightly improve the community network as produced by the DREAM5 challenge.

### Comparing Netter to similar techniques

In this subsection, we will compare Netter with other post-processing approaches for GRN inference predictions and other algorithms that incorporate global network information in their inference process. We are not aware of any other methods that use structural properties of the output network to guide the inference prediction on a large scale. However, as discussed in the introduction, both CLR and ARACNE can be considered as extensions of relevance networks which correct the mutual information (MI) scores using a more global network context. Network Deconvolution and the Silencer on the other hand are post-processing techniques that attempt to separate direct causal effects from indirect effects and have been applied for GRN inference. As mentioned in the introduction, WGCNA raises a pairwise correlation matrix to a certain power to maximally fit the scale-free topology measure. Although, the idea is similar to Netter, both methods cannot be compared directly. WCGNA only changes the edge weight values but does not change the ranking of edges. As baseline for our comparison, we infer networks by calculating MI scores for each pair of genes. Next, we also infer the networks using ARACNE and CLR. For each network, we post-process these three predictions using Netter, Network Deconvolution and the Silencer. This results in twelve different predictions for each network. We use the same full dataset as in the performance tests. Again we use the AUROC and AUPR scores as evaluation metrics, however we do not adopt the pre-processing procedure described in the ‘Selected data sets and evaluation measures’ subsection, as we are interested in comparing between methods as opposed to relative gains in this test.

**Fig. 6:**
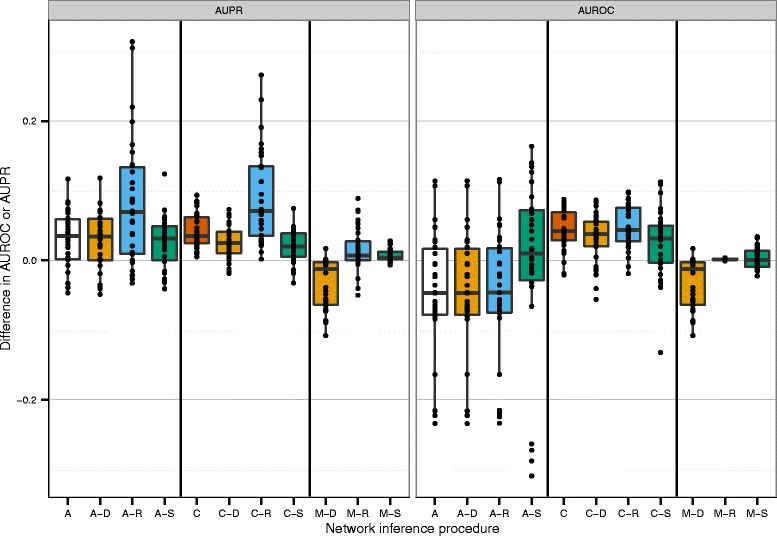
Performance comparison of Netter to similar (post-procesing) algorithms. (A = ARACNE, C = CLR, M = Mutual information, R = Netter re-ranking, D = Network Deconvolution, S = Silencer. ’–’ indicates post-processing. The MI prediction is used as a baseline and the relative difference in AUPR and AUROC of the complete ranking of the other predictions is plotted. Each dot represents a single network prediction

Figure 6 shows the change in evaluation metric compared to the MI prediction. Each dot resembles a final network prediction. In total 11 boxplots are shown, two include ARACNE and CLR predictions without further post-processing. The remaining nine are post-processed networks of the mutual information, ARACNE and CLR predictions using Netter, the Silencer or Network Deconvolution.

The figure shows that the ARACNE method has a higher AUPR score compared to the MI network but at the cost of a decreased AUROC score. This is caused by ARACNE setting a large number of interactions to 0, a more aggressive approach than most other algorithms. CLR both has higher AUROC and AUPR scores than the original MI prediction. These performance gains are to be expected, as both algorithms are widely adopted and have been successfully applied to various use cases. Among the post-processing algorithms, Netter is the clearly the most successful one. Applying Netter results in a substantial improvement for the AUPR score of the ARACNE and CLR predictions as also shown in the previous subsections and a small improvement in AUPR score for the MI network. The smaller gain for the MI network can be explained by the lower accuracy of the initial prediction, as we will further discuss in the following subsection. Netter does not seem to influence the AUROC score of the MI, ARACNE or CLR predictions. This is because Netter is a conservative approach, only re-ranking the first *x* (*in casu* 750), allowing no new links to enter the prediction. Applying Network Deconvolution results in a decrease in AUROC and AUPR in all but a few cases for the MI prediction. It has no effect on the ARACNE predictions and lowers the prediction accuracy of CLR in general. The Silencer is able to correct the loss in AUROC score originating from ARACNE but does not have a positive effect in all other cases. The performance of the Silencer has been subject to controversy [34]. Concluding, we believe that Netter compares favorably to other post-processing approaches. Furthermore it has the advantage that it is not limited to correlation-like measures but can be applied to rankings or ranking ensembles of different algorithms.

### Characteristics of improvement with regard to the initial prediction accuracy

**Fig. 7:**
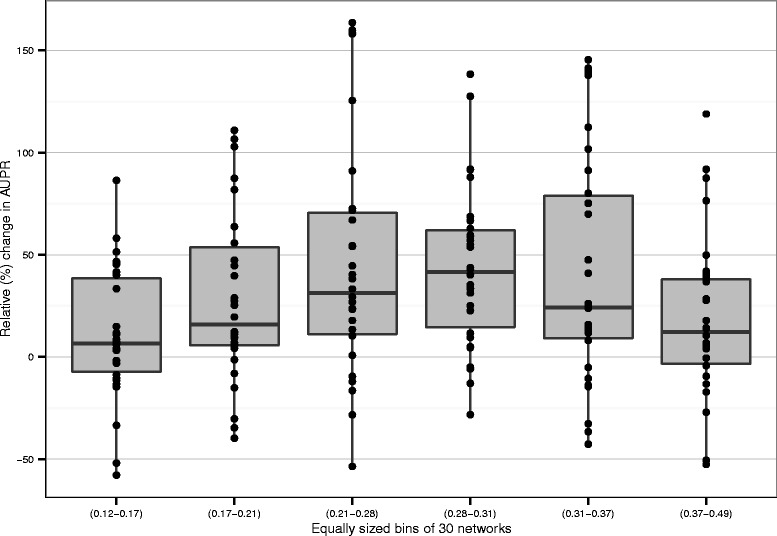
Characteristics of improvement with regard to the initial prediction accuracy. Relative (%) change in AUPR of the full dataset is plotted, binned in equally sized groups of 30 networks. In general, Netter’s potential to improve the prediction is higher when the initial prediction is more accurate

Figure 7 shows the results of the performance tests in a different way. We grouped the 180 re-rankings into 6 equally sized bins using the accuracy of the initial prediction as binning criteria. The y-axis shows the relative gain in AUPR compared to the original prediction (e.g. an original AUPR score of 0.20 which is increased to 0.40 by applying Netter would be plotted at a y-value of 100 %). For each bin, the means of the boxplot are well above the baseline of 0 and in less than 30 % of the cases the performance is lowered. Furthermore, higher gains (up to 150 %) are achieved as opposed to less accurate re-rankings (maximum at −50 %). The potency of Netter to improve predictions is at its lowest for predictions which are the least accurate to start with. This makes sense, as Netter relies on the accuracy of the initial prediction to guide its re-ranking process in the right direction. We see a general trend that applying Netter becomes increasingly interesting up to a certain level if the initial accuracy of the prediction is higher. The majority of predictions with the highest initial accuracy have a lower mean improvement, although some of the most accurate initial predictions can still be substantially improved by applying Netter.

### Successive applications of Netter

**Fig. 8:**
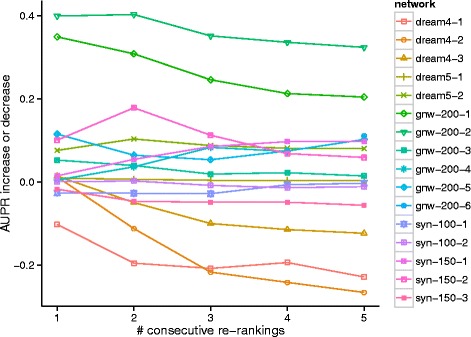
Evolution of the performance during consecutive applications of Netter. Netter is consecutively applied using default setting and penalty functions on the reduced test dataset. The performance increase or decrease compared to the original prediction is plotted after each re-ranking

Netter can also be applied on the outcome of a previous Netter re-ranking. Figure 8 shows the evolution of the AUPR score of chaining Netter on the reduced test dataset of 15 GENIE3 predictions. A second re-ranking procedure has a mixed effect on the performance, with about as many networks improving in accuracy as predictions becoming less accurate. Further successive applications of Netter result in an accuracy loss in the general case although many networks continue to show an improvement compared to original ranking after 5 re-rankings. The obtained accuracy is comparable to running Netter with increasingly less stringent regularization penalty (divergence cost function) as the influence of the original ranking is decreased with every re-ranking.

### Parameter and structure cost function stability analysis

The large number of parameters which can be set in Netter raises the questions of how one can tune these parameters and how influential these parameters are on the prediction accuracy. Furthermore, one needs to be sure that a small change in the definition of the structural functions does not lead to a large change in the re-ranking accuracy. To address the first question, Netter is equipped with a logger system which can track among others the prediction accuracy, the total cost function, the individual penalty functions and the accept/revert ratio of the simulated annealing process at desired intervals.

To address the second question, first the performance tests used a large and diverse dataset: including benchmark data and networks of different dimensions, created by two different simulators to decrease the change of obtaining inflated figures by chance. Secondly, we have performed parameter sweeps by changing the value of one parameter and keeping the other constant. Thirdly, we substituted the default structural cost function mapping for each penalty with three times sixteen other simple structural cost functions with different slopes and intersects by varying *a* and *b*. Table 3 lists the default parameter settings and explores different values for the structural penalty functions, the balance factor *α*, the subnetwork size parameter *n* and associated coefficients *π*_*i*_. The tables shows the average AUPR over all 15 networks, the individual values can be found in the Additional files 1–4. We discuss the parameter settings and the results of the stability tests in the following subsections.

**Table 3:** Stability tests of *α*, *n*, *π*_*i*_ and the relative weights of the structural penalties

Default setting *n*=25,*π*_*i*_=0.5i	*n*=50,*π*_*i*_=0.5i	*n*=75,*π*_*i*_=0.5i	*π*_*i*_=0.25i, *n*=50	*π*_*i*_=3i, *n*=50
0.41	0.41	0.41	0.41	0.42
Default setting *α*=10^−5^	*α*=10^−2^	*α*=10^−3^	*α*=10^−4^	*α*=10^−6^
0.41	0.37	0.40	0.41	0.42
Default setting *g*4=2.0	*g*4=0.0	*g*4=1.0	*g*4=5.0	*g*4=10.0
0.41	0.39	0.41	0.41	0.41
Default setting *r*=25.0	*r*=0.0	*r*=1.0	*r*=15.0	*g*=35.0
0.41	0.36	0.38	0.41	0.41
Default setting *a*=25.0	*a*=0.0	*a*=50.0	*a*=75.0	*a*=100.0
0.41	0.40	0.41	0.41	0.41

The average AUPR score on a subset of 15 GENIE3 predictions is shown and compared to the score using default settings. Parameters not listed were set to default values. (g4 = graphlet, r = regulatory, a = anti-dominating)

#### Influence of the number of optimization runs on the convergence of Netter

**Fig. 9:**
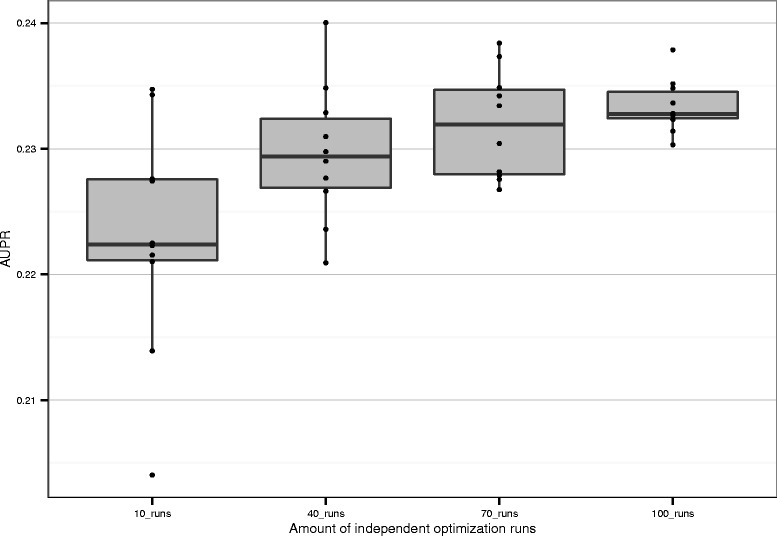
Influence of the number of optimization runs on the convergence of Netter. Netter is run ten times with a varying number of independent optimization runs (10, 40, 70, 100). Each dot represents the AUPR of the re-ranked prediction

Netter runs a number of independent optimization runs before averaging and producing the final output ranking. We have shown that using this ensemble method, the output of Netter is robust if the same settings are used. We further explore the stability of Netter with regard to a variable number of independent optimization runs. Figure 9 shows 10 runs of Netter using 10, 40, 70 and 100 independent runs before averaging on the *E.coli.* DREAM5 network. All other networks show similar behaviour. It shows that the mean performance gain increases if more optimization runs are performed. The variance between the final re-ranking also decreases with an increasing amount of optimization runs. However, the mean performance difference between 10 runs and 100 runs is only 0.01, while the difference with the original ranking evalation is 0.08. Therefore, if computing power is a bottleneck and many networks need to be re-ranked, a reduced number of optimization runs can be used without a large loss in accuracy.

#### Influence of the subnetwork size *n* and coefficients *π*_*i*_

When calculating the structural cost function, the ranking is divided into subnetworks of increasing size. The size is determined by the parameter *n* and the impact on the total structural cost function of a single subnetwork *g*_*i*_ is determined by the associated coefficient *π*_*i*_. Increasing the subnetwork size will decrease the computation time, as there are fewer subnetworks of which the structural properties need to be tracked. On the other hand, a larger subnetwork size leads to less structural differentiation options for the different links, possibly resulting in a lower accuracy. Table 3 shows the results for varying *n* and *π*_*i*_. The performance is stable with regard to the coefficient choice for *π*_*i*_ and the subnetwork size *n* over a wide range of values. Concluding, we recommend to set *n* to a small value (e.g. 25 or 1/30 of *x*) to ease the computational demands but to allow for maximum differentiation, however the choice of *n* and *π*_*i*_ is not crucial with regard to the performance.

#### Influence of varying the global balance factor *α*

Probably the most important parameter in the re-ranking algorithm is the parameter *α* which determines the trade-off between the divergence cost and structural cost of a ranking. If this parameter is set too high, the algorithm will not allow any changes to be made to the original ranking. Whereas if the parameter is set too low, the re-ranking process will not use the original ranking to guide the optimization process. We vary this parameter by setting the values 10^−*i*^, with *i*=2…6. The results are shown in Table 3. For high values of *α*, the network will only allow small changes to the network, resulting in accuracy which is between the accuracy of the original prediction and the maximum accuracy which can be achieved after re-ranking. Interestingly, the accuracy seems to be stable for the values *i*=4…6. We believe this is due to the ensemble approach in which we average over several optimization processes.

#### Influence of varying the relative weight of a individual structure penalty function

The impact of the individual penalty functions on the total structural cost function can be adjusted by changing the associated weights of each penalty function. These weights are typically set by running the algorithm several times with some initial settings and by tracking the individual penalty scores using the logging system. The influence of these parameters is shown in Table 3. For all three penalty functions, a performance loss can be seen if the penalty influence is set to zero and as such is not included in the structural cost function. The weight of all three penalties is shown to be robust for a wide range of values, meaning that a small change in this weight does not result in a big effect on the outcome. As a rule of thumb, we suggest that the weights are set using the logger system to values such that all penalties which the user designed and included more or less equally contribute to the decrease in the overall penalty function. This way, the weights of the individual penalty functions seem to have little effect on the accuracy increase of the re-ranking process.

#### Influence of the individual structure cost penalty mappings

In order to test the robustness, we replaced the default v-shaped function (*f*(*y*)=∥*ay*+*b*∥) of each structural penalty in a 4 by 4 grid search. *b* was set such that the function had zero cost at different values for the structural property *y* and for each setting of *b*, four different slopes were selected by varying *a*. Additional files 1–3 contain the exact values of *a* and *b*, a visualization of the functions and the performance metrics of the networks re-ranked by Netter using these settings. For the graphlet based and the gene limiting penalty, the decrease in average AUPR over the 15 networks was at most 0.02 and corresponded to the setting in which the penalty function was moved furthest from the original intersect. We therefore conclude that these penalties are stable over a wide range of possible mapping definitions. The anti-dominate penalty showed a slightly faster decrease in AUPR if the intersection with the x-axis was moved further to the right. In the extreme case the performance dropped to 0.38 from 0.41. The performance loss is slightly more pronounced because unlike the latter penalties the penalty cost associated with *y*-values left of the intersect have no meaning, as it does not make sense to discourage rankings which explore different regions of the network. Concluding, the exact shape of all three structural penalty functions is robust and only decreases slowly if the function is moved closer to the inverse function. The individual network re-ranking scores can be found in Additional files 1–3.

### Further exploration of the impact of the structural penalty function definition

In addition to the tests in the previous subsection in which we varied the shape and the relative performance of the structural penalty functions, we believe it also important to investigate how Netter behaves in extreme settings. The goal is to both establish some baselines for the performance metrics and to help gain intuition about the presented performance and stability results. In a first test, we excluded all structural penalties and the divergence cost function and again re-ranked the reduced subset of 15 networks. The simulated annealing scheme was altered to accept every proposed ranking. This results in randomly shuffling the ranking for a set number of iterations before averaging the obtained rankings. Table 4 shows the AUPR results for 300, 3000 and the default value of 30,000 iterations averaged over the standard value of 100 independent optimization runs.

**Table 4:** AUPR results of re-ranking without penalty functions for a set number of iterations

Initial	Default re-rank.	300 iter.	3000 iter.	30,000 iter.
0.34	0.41	0.34 (±0.01)	0.31 (±0.02)	0.21 (±0.02)

Average values over 10 runs are shown on the reduced test dataset. Standard deviation is listed between brackets

This experiment was repeated 10 times and the standard deviation between runs is shown between brackets. The table shows that the performance drops as the number of iterations increases. This is expected, as the initial prediction is more confident about the top of the ranking which would as a result contain more true positive links. Randomly shuffling the ranking would eventually lead to a uniform distribution of the true positive links, resulting in a worse AUPR score and an AUROC score of 0.5. Due to the ensemble nature of Netter, the standard deviation of the performance loss between the final obtained rankings remains small, although the obtained ranking diverges more than in the latter case.

In a second test, we modify the structural penalties such that they attempt to optimize the inverse function. For the regulatory gene limiting penalty and the graphlet-based penalty this is achieved by changing the v-shaped function intercept to 1−*b*. The optimization process will then attempt to lower the amount of G4 graphlets and increase the numbers of nodes with outgoing edges. We excluded the anti-dominating penalty from these experiments, as the inverse of this function is not well defined. Table 5 lists the average AUPR score over the subset of 15 networks.

**Table 5:** AUPR results of re-ranking with the inverse of the default structural properties

Initial	Default re-rank.	Inv. graphlet	Inv. regulatory	Both inv.
0.34	0.41	0.32	0.30	0.26

Even in the extreme case in which one uses two inverted functions which are clearly not typical for a gene regulatory network, the accuracy of the prediction remains higher than the randomly shuffled network. This is due to the divergence cost function which attempts to keep the new ranking as close as possible to the original. In case only one inverted function is used, the performance loss is less pronounced, suggesting that other structural properties can counter the effects of ill-chosen penalty functions to some extent. Overall we believe that the performance gain is promising if well-motivated structural properties are used and the performance gain is robust to the exact transformation of the structural property into a penalty function.

## Conclusions

In this work we presented Netter, a novel post-processing algorithm for gene regulatory network predictions. Our algorithm re-ranks a sorted list of predicted regulatory interactions using known structural properties of the network. The algorithm works by defining an optimization problem in which we minimize a weighted sum of desired structural properties and a regularization term penalizing divergence from the original prediction. This optimization problem is solved several times using simulated annealing, after which the obtained networks are aggregated using average rank to obtain the final output. We offer a flexible system in which desired structural properties can be developed and included. Expert users can tune the system to include specific prior knowledge but we show that by using three suggested more general penalty functions we can obtain a large accuracy gain on benchmark and artificial data. Using these settings Netter outperforms other post-processing methods such as the Silencer and Network Deconvolution. Although our method is heavily parameterized, we have shown that the performance increase is robust for a wide range of values and structural cost penalty functions. Furthermore, especially the top of the ranking is improved by Netter, making our method appealing for practical use. Finally, we have shown that Netter can further improve the DREAM5 community prediction of the *E.coli.* network inferred from real expression data.

## Additional files

Additional file 1
**Add 1 Graphlet Penalty.pdf.** Additional file 1 is a pdf file containing the default mapping of the graphlet based penalty and a detailed view of the stability tests. (PDF 362 KB)

Additional file 2
**Add 2 Regulatory Penalty.pdf.** Additional file 2 is a pdf file containing the default mapping of the regulatory gene penalty and a detailed view of the stability tests. (PDF 411 KB)

Additional file 3
**Add 3 AntiDominating Penalty.pdf.** Additional file 3 is a pdf file containing the default mapping of the anti-dominating penalty and a detailed view of the stability tests. (PDF 411 KB)

Additional file 4
**Add 4 Performance tests individual results.xlsx.** Additional file 4 is an xlsx file containing the detailed results of all performance tests. (XLSX 295 KB)
