# Supplementary material for: Netter: re-ranking gene network inference predictions using structural network properties
Source: BMC Bioinformatics. 2016 Feb 9;17:76. doi: 10.1186/s12859-016-0913-0 (PMC4746913; doi:10.1186/s12859-016-0913-0)
Supplement: Additional file 1 — Add 1 Graphlet Penalty.pdf. Additional file 1 is a pdf file containing the default mapping of the graphlet based penalty and a detailed view of the stability tests. (PDF 362 KB) [file 12859_2016_913_MOESM1_ESM.pdf]

# Graphlet-G4 cost function shape variability analysis

We investigate how the definition of the cost function influences the performance on a randomly reduced subset of 15 networks. The default penalty mapping is replaced by 15 different simple penalty mappings as shown below. All other parameters and penalty mappings were set to the default values. The AUPR scores are shown and compared to the scores obtained by using the default settings.

## Default penalty function definition

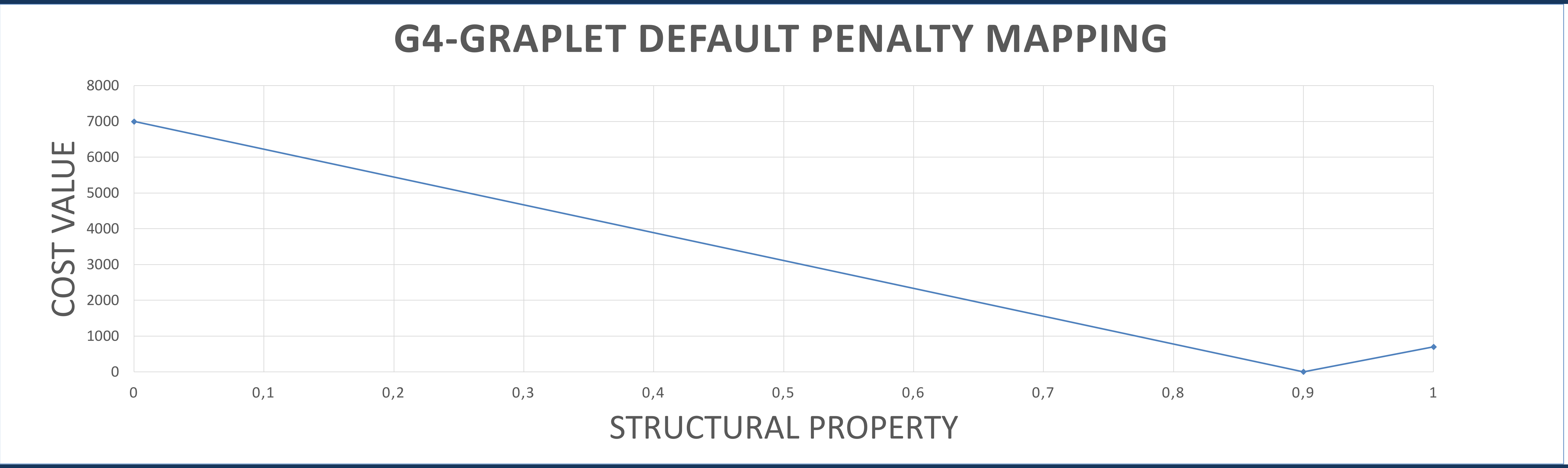


## Stability testing - Visualization of the grid search functions

We visualize the other G4-graphlet penalty mappings which were used in the 4 by 4 grid search in the stability tests.

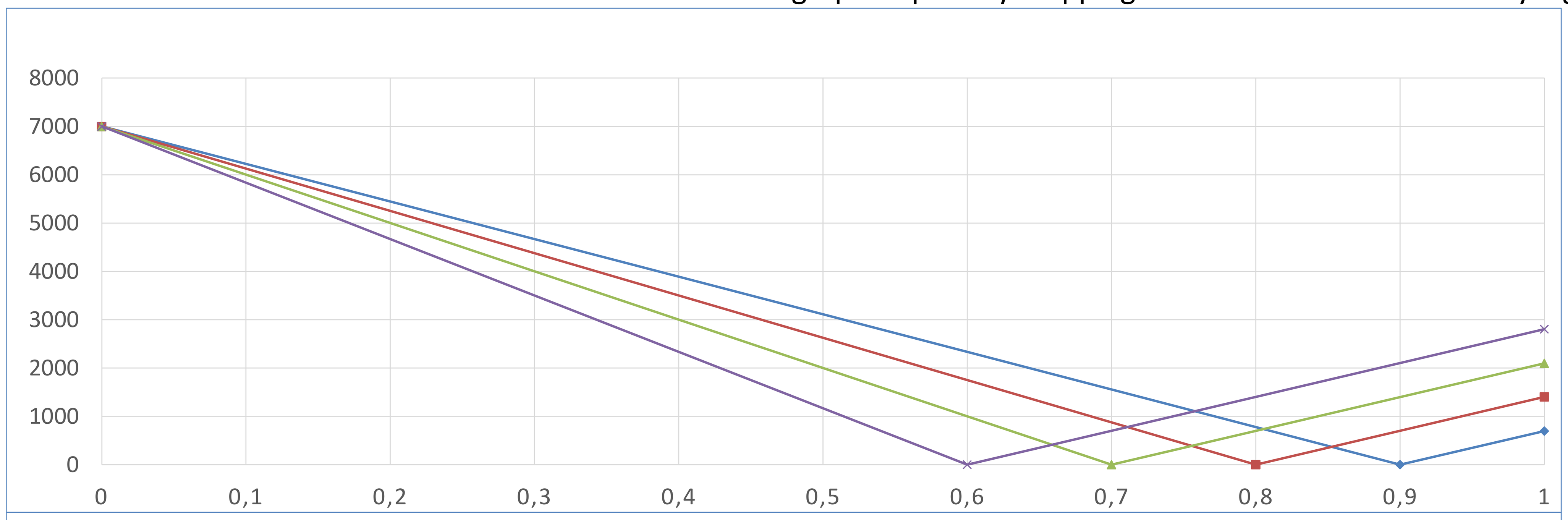

DEFAULT

| 1 | | 2 | | 3 | | 4 | |
|---|---|---|---|---|---|---|---|
| Key Value | Cost | Key Value | Cost | Key Value | Cost | Key Value | Cost |
| 0 | 7000 | 0 | 7000 | 0 | 7000 | 0 | 7000 |
| 0,9 | 0 | 0,8 | 0 | 0,7 | 0 | 0,6 | 0 |
| 1 | 700 | 1 | 1400 | 1 | 2100 | 1 | 2800 |

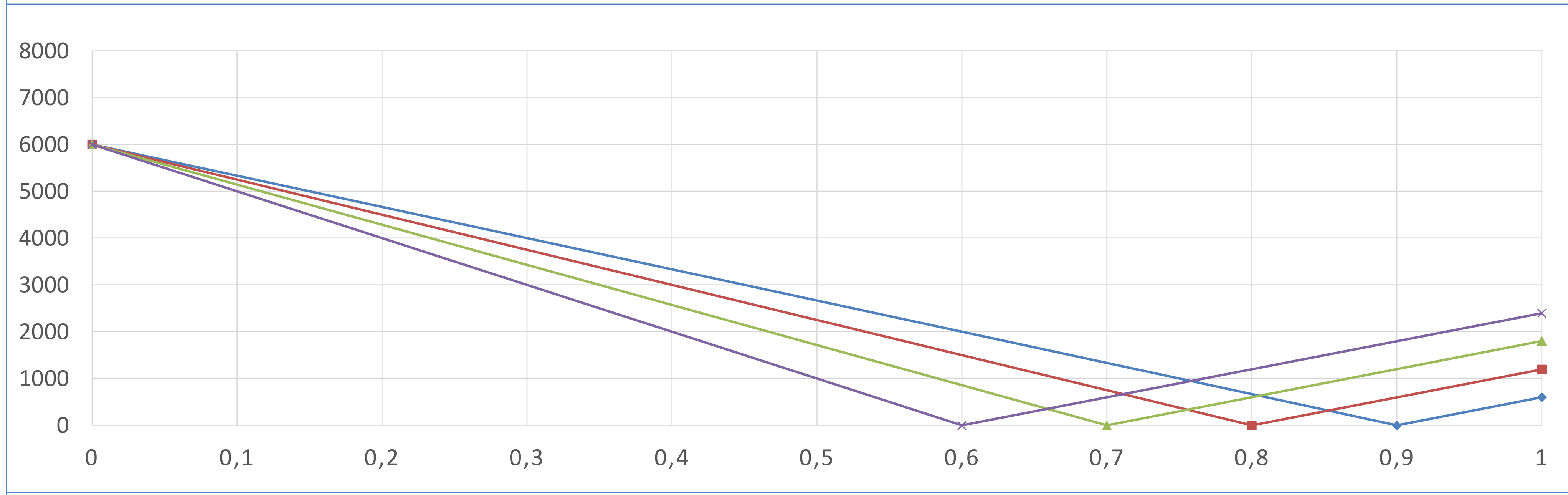

| 5 | | 6 | | 7 | | 8 | |
|---|---|---|---|---|---|---|---|
| Key Value | Cost | Key Value | Cost | Key Value | Cost | Key Value | Cost |
| 0 | 6000 | 0 | 6000 | 0 | 6000 | 0 | 6000 |
| 0,9 | 0 | 0,8 | 0 | 0,7 | 0 | 0,6 | 0 |
| 1 | 600 | 1 | 1200 | 1 | 1800 | 1 | 2400 |

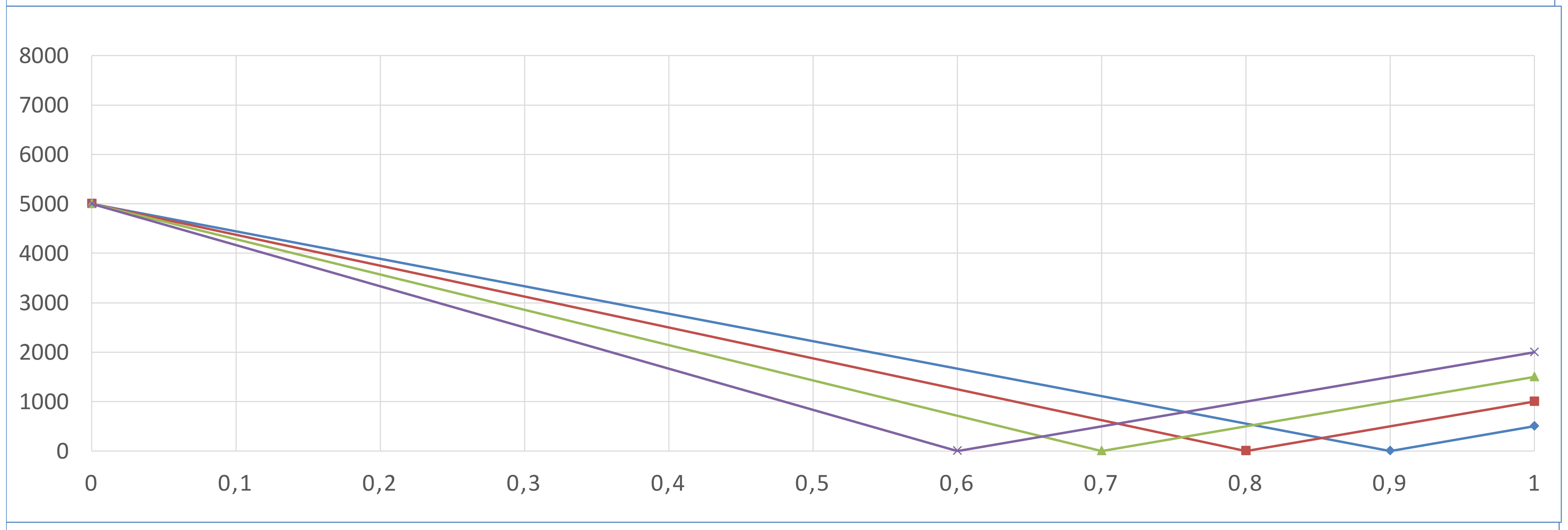

| 9 | | 10 | | 11 | | 12 | |
|---|---|---|---|---|---|---|---|
| Key Value | Cost | Key Value | Cost | Key Value | Cost | Key Value | Cost |
| 0 | 5000 | 0 | 5000 | 0 | 5000 | 0 | 5000 |
| 0,9 | 0 | 0,8 | 0 | 0,7 | 0 | 0,6 | 0 |
| 1 | 500 | 1 | 1000 | 1 | 1500 | 1 | 2000 |

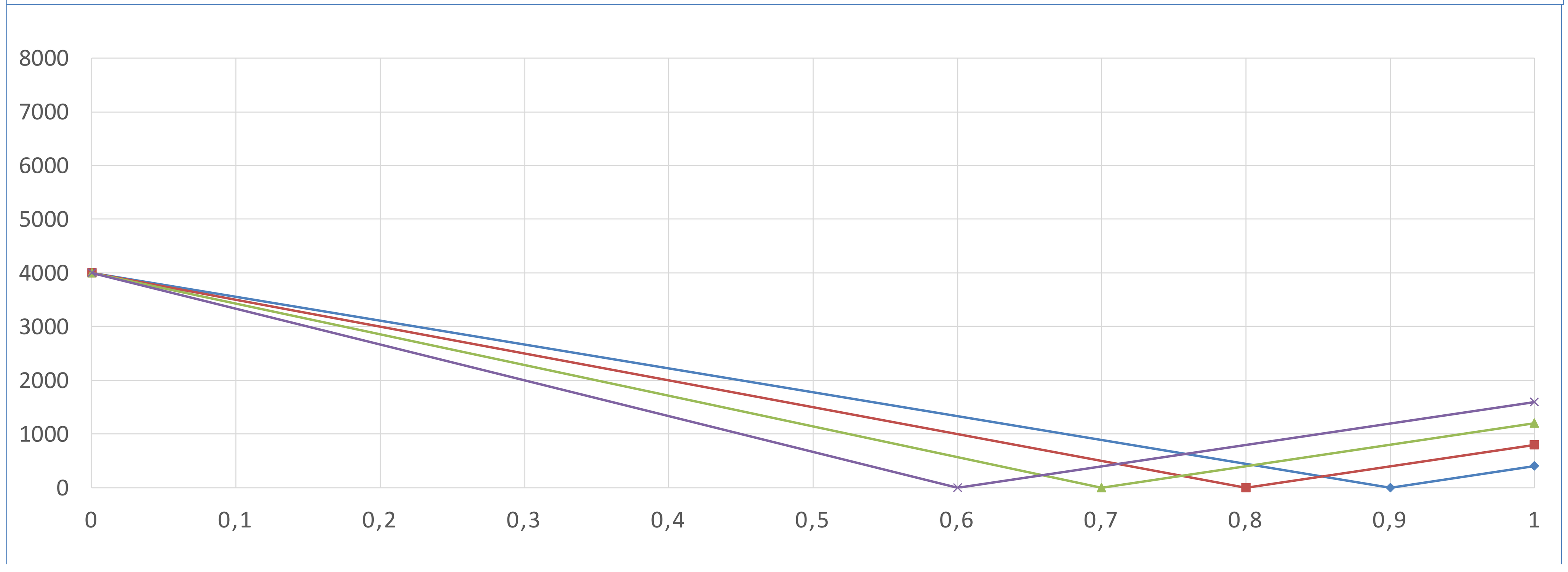

| 13 | | 14 | | 15 | | 16 | |
|---|---|---|---|---|---|---|---|
| Key Value | Cost | Key Value | Cost | Key Value | Cost | Key Value | Cost |
| 0 | 4000 | 0 | 4000 | 0 | 4000 | 0 | 4000 |
| 0,9 | 0 | 0,8 | 0 | 0,7 | 0 | 0,6 | 0 |
| 1 | 400 | 1 | 800 | 1 | 1200 | 1 | 1600 |

# Stability testing - Indivudual re-ranking AUPR scores

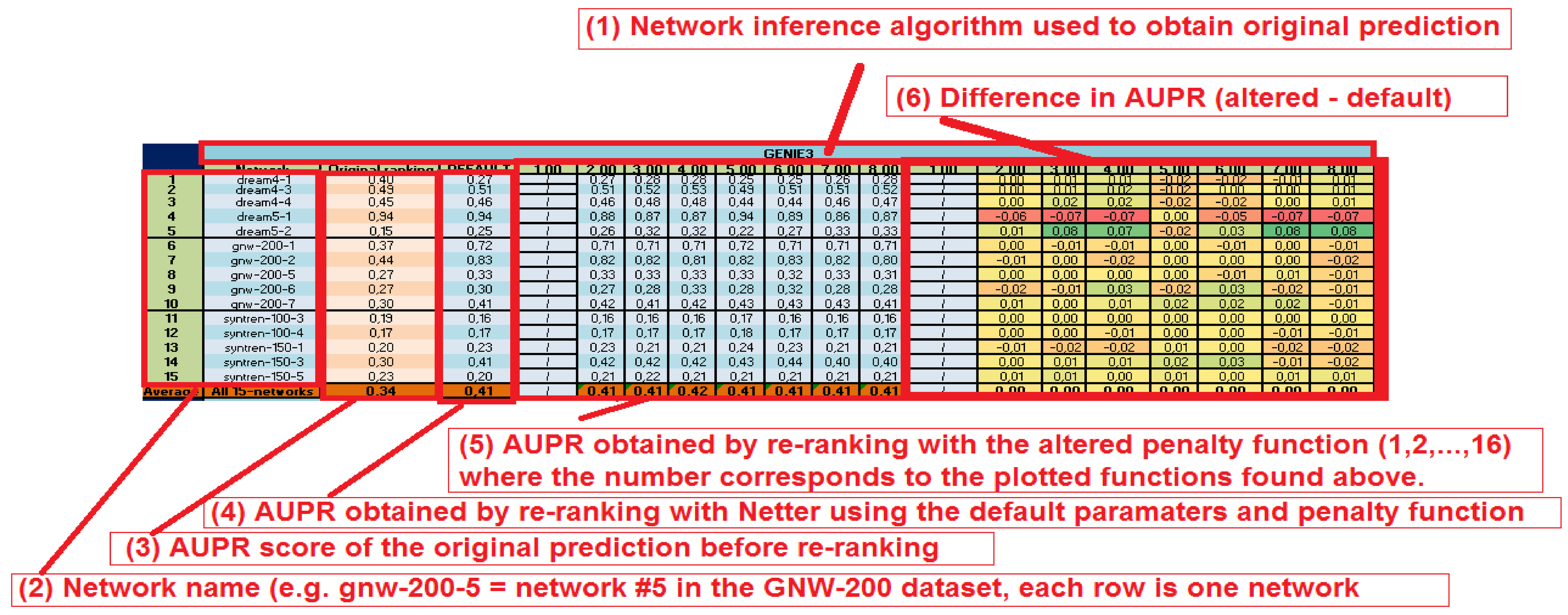


The table below lists the AUPR evaluation of the original prediction, the re-ranked prediction using default settings and all re-rankings obtained by using one of the altered penalty mappings found above. Each row is a network, each column is a re-ranking using a different setting.

| | GENIE3 | | | | | | | | | | | | | | | | | | |
|---|---|---|---|---|---|---|---|---|---|---|---|---|---|---|---|---|---|---|---|
| | Network | Original ranking | DEFAULT | 1,00 | 2,00 | 3,00 | 4,00 | 5,00 | 6,00 | 7,00 | 8,00 | 1,00 | 2,00 | 3,00 | 4,00 | 5,00 | 6,00 | 7,00 | 8,00 |
| 1 | dream4-1 | 0,40 | 0,27 | 0,27 | 0,27 | 0,28 | 0,28 | 0,25 | 0,25 | 0,26 | 0,28 | / | 0,00 | 0,01 | 0,01 | -0,02 | -0,02 | -0,01 | 0,01 |
| 2 | dream4-3 | 0,49 | 0,51 | 0,51 | 0,51 | 0,52 | 0,53 | 0,49 | 0,51 | 0,51 | 0,52 | / | 0,00 | 0,01 | 0,02 | -0,02 | 0,00 | 0,00 | 0,01 |
| 3 | dream4-4 | 0,45 | 0,46 | 0,46 | 0,46 | 0,48 | 0,48 | 0,44 | 0,44 | 0,46 | 0,47 | / | 0,00 | 0,02 | 0,02 | -0,02 | -0,02 | 0,00 | 0,01 |
| 4 | dream5-1 | 0,94 | 0,94 | 0,94 | 0,88 | 0,87 | 0,87 | 0,94 | 0,89 | 0,86 | 0,87 | / | -0,06 | -0,07 | -0,07 | 0,00 | -0,05 | -0,07 | -0,07 |
| 5 | dream5-2 | 0,15 | 0,25 | 0,25 | 0,26 | 0,32 | 0,32 | 0,22 | 0,27 | 0,33 | 0,33 | / | 0,01 | 0,08 | 0,07 | -0,02 | 0,03 | 0,08 | 0,08 |
| 6 | gnw-200-1 | 0,37 | 0,72 | 0,72 | 0,71 | 0,71 | 0,71 | 0,72 | 0,71 | 0,71 | 0,71 | / | 0,00 | -0,01 | -0,01 | 0,00 | -0,01 | 0,00 | -0,01 |
| 7 | gnw-200-2 | 0,44 | 0,83 | 0,83 | 0,82 | 0,82 | 0,81 | 0,82 | 0,83 | 0,82 | 0,80 | / | -0,01 | 0,00 | -0,02 | 0,00 | 0,00 | 0,00 | -0,02 |
| 8 | gnw-200-5 | 0,27 | 0,33 | 0,33 | 0,33 | 0,33 | 0,33 | 0,33 | 0,32 | 0,33 | 0,31 | / | 0,00 | 0,00 | 0,00 | 0,00 | -0,01 | 0,01 | -0,01 |
| 9 | gnw-200-6 | 0,27 | 0,30 | 0,30 | 0,27 | 0,28 | 0,33 | 0,28 | 0,32 | 0,28 | 0,28 | / | -0,02 | -0,01 | 0,03 | -0,02 | 0,03 | -0,02 | -0,01 |
| 10 | gnw-200-7 | 0,30 | 0,41 | 0,41 | 0,42 | 0,41 | 0,42 | 0,43 | 0,43 | 0,43 | 0,41 | / | 0,01 | 0,00 | 0,01 | 0,02 | 0,02 | 0,02 | -0,01 |
| 11 | syntren-100-3 | 0,19 | 0,16 | 0,16 | 0,16 | 0,16 | 0,16 | 0,17 | 0,16 | 0,16 | 0,16 | / | 0,00 | 0,00 | 0,00 | 0,00 | 0,00 | 0,00 | 0,00 |
| 12 | syntren-100-4 | 0,17 | 0,17 | 0,17 | 0,17 | 0,17 | 0,17 | 0,18 | 0,17 | 0,17 | 0,17 | / | 0,00 | 0,00 | -0,01 | 0,00 | 0,00 | -0,01 | -0,01 |
| 13 | syntren-150-1 | 0,20 | 0,23 | 0,23 | 0,23 | 0,21 | 0,21 | 0,24 | 0,23 | 0,21 | 0,21 | / | -0,01 | -0,02 | -0,02 | 0,01 | 0,00 | -0,02 | -0,02 |
| 14 | syntren-150-3 | 0,30 | 0,41 | 0,41 | 0,42 | 0,42 | 0,42 | 0,43 | 0,44 | 0,40 | 0,40 | / | 0,00 | 0,01 | 0,01 | 0,02 | 0,03 | -0,01 | -0,02 |
| 15 | syntren-150-5 | 0,23 | 0,20 | 0,20 | 0,21 | 0,22 | 0,21 | 0,21 | 0,21 | 0,21 | 0,21 | / | 0,01 | 0,01 | 0,00 | 0,01 | 0,00 | 0,01 | 0,01 |
| Average | All 15-networks | 0,34 | 0,41 | 0,41 | 0,41 | 0,41 | 0,42 | 0,41 | 0,41 | 0,41 | 0,41 | / | 0,00 | 0,00 | 0,00 | 0,00 | 0,00 | 0,00 | 0,00 |

| | GENIE3 | | | | | | | | | | | | | | | | | | |
|---|---|---|---|---|---|---|---|---|---|---|---|---|---|---|---|---|---|---|---|
| | Network | Original ranking | DEFAULT | 9,00 | 10,00 | 11,00 | 12,00 | 13,00 | 14,00 | 15,00 | 16,00 | 9,00 | 10,00 | 11,00 | 12,00 | 13,00 | 14,00 | 15,00 | 16,00 |
| 1 | dream4-1 | 0,40 | 0,27 | 0,24 | 0,24 | 0,26 | 0,27 | 0,26 | 0,25 | 0,23 | 0,27 | -0,03 | -0,03 | -0,01 | 0,00 | -0,01 | -0,02 | -0,04 | 0,00 |
| 2 | dream4-3 | 0,49 | 0,51 | 0,51 | 0,51 | 0,52 | 0,52 | 0,52 | 0,49 | 0,50 | 0,49 | 0,00 | 0,00 | 0,01 | 0,01 | 0,01 | -0,02 | -0,01 | -0,02 |
| 3 | dream4-4 | 0,45 | 0,46 | 0,47 | 0,46 | 0,46 | 0,46 | 0,47 | 0,45 | 0,44 | 0,48 | 0,00 | 0,00 | 0,00 | 0,00 | 0,00 | -0,01 | -0,02 | 0,01 |
| 4 | dream5-1 | 0,94 | 0,94 | 0,95 | 0,88 | 0,87 | 0,87 | 0,94 | 0,89 | 0,88 | 0,88 | 0,01 | -0,06 | -0,06 | -0,07 | 0,00 | -0,05 | -0,06 | -0,06 |
| 5 | dream5-2 | 0,15 | 0,25 | 0,23 | 0,26 | 0,34 | 0,33 | 0,22 | 0,25 | 0,34 | 0,33 | -0,02 | 0,02 | 0,09 | 0,09 | -0,02 | 0,00 | 0,09 | 0,09 |
| 6 | gnw-200-1 | 0,37 | 0,72 | 0,70 | 0,72 | 0,72 | 0,72 | 0,72 | 0,71 | 0,71 | 0,69 | -0,01 | 0,01 | 0,00 | 0,00 | 0,01 | 0,00 | 0,00 | -0,02 |
| 7 | gnw-200-2 | 0,44 | 0,83 | 0,83 | 0,82 | 0,83 | 0,82 | 0,83 | 0,83 | 0,82 | 0,81 | 0,00 | -0,01 | 0,01 | -0,01 | 0,00 | 0,01 | -0,01 | -0,01 |
| 8 | gnw-200-5 | 0,27 | 0,33 | 0,32 | 0,32 | 0,32 | 0,32 | 0,33 | 0,33 | 0,33 | 0,31 | -0,01 | -0,01 | 0,00 | -0,01 | 0,00 | 0,00 | 0,01 | -0,02 |
| 9 | gnw-200-6 | 0,27 | 0,30 | 0,28 | 0,27 | 0,26 | 0,32 | 0,27 | 0,26 | 0,27 | 0,28 | -0,01 | -0,03 | -0,03 | 0,02 | -0,02 | -0,03 | -0,02 | -0,02 |
| 10 | gnw-200-7 | 0,30 | 0,41 | 0,43 | 0,43 | 0,42 | 0,40 | 0,42 | 0,42 | 0,43 | 0,41 | 0,02 | 0,02 | 0,00 | -0,01 | 0,00 | 0,01 | 0,02 | -0,01 |
| 11 | syntren-100-3 | 0,19 | 0,16 | 0,16 | 0,17 | 0,16 | 0,16 | 0,16 | 0,17 | 0,17 | 0,17 | 0,00 | 0,01 | 0,00 | 0,00 | 0,00 | 0,01 | 0,01 | 0,00 |
| 12 | syntren-100-4 | 0,17 | 0,17 | 0,18 | 0,17 | 0,17 | 0,17 | 0,17 | 0,18 | 0,18 | 0,17 | 0,01 | 0,00 | 0,00 | 0,00 | 0,00 | 0,01 | 0,01 | 0,00 |
| 13 | syntren-150-1 | 0,20 | 0,23 | 0,26 | 0,23 | 0,23 | 0,22 | 0,22 | 0,26 | 0,24 | 0,24 | 0,03 | 0,00 | 0,00 | -0,01 | -0,01 | 0,03 | 0,01 | 0,01 |
| 14 | syntren-150-3 | 0,30 | 0,41 | 0,44 | 0,45 | 0,43 | 0,43 | 0,43 | 0,43 | 0,45 | 0,42 | 0,02 | 0,04 | 0,02 | 0,02 | 0,02 | 0,02 | 0,04 | 0,01 |
| 15 | syntren-150-5 | 0,23 | 0,20 | 0,20 | 0,20 | 0,21 | 0,21 | 0,21 | 0,21 | 0,21 | 0,21 | 0,00 | 0,00 | 0,01 | 0,00 | 0,00 | 0,00 | 0,01 | 0,00 |
| Average | All 15-networks | 0,34 | 0,41 | 0,41 | 0,41 | 0,41 | 0,41 | 0,41 | 0,41 | 0,41 | 0,41 | 0,00 | 0,00 | 0,00 | 0,00 | 0,00 | 0,00 | 0,00 | 0,00 |

# Stability testing - Varying the relative weighing parameter variability analysis

We investigate what the effect is of making the penalty more or less important in the total cost function. This is done by varying the coefficient on a randomly reduced subset of 15 networks. AUPR values are shown.

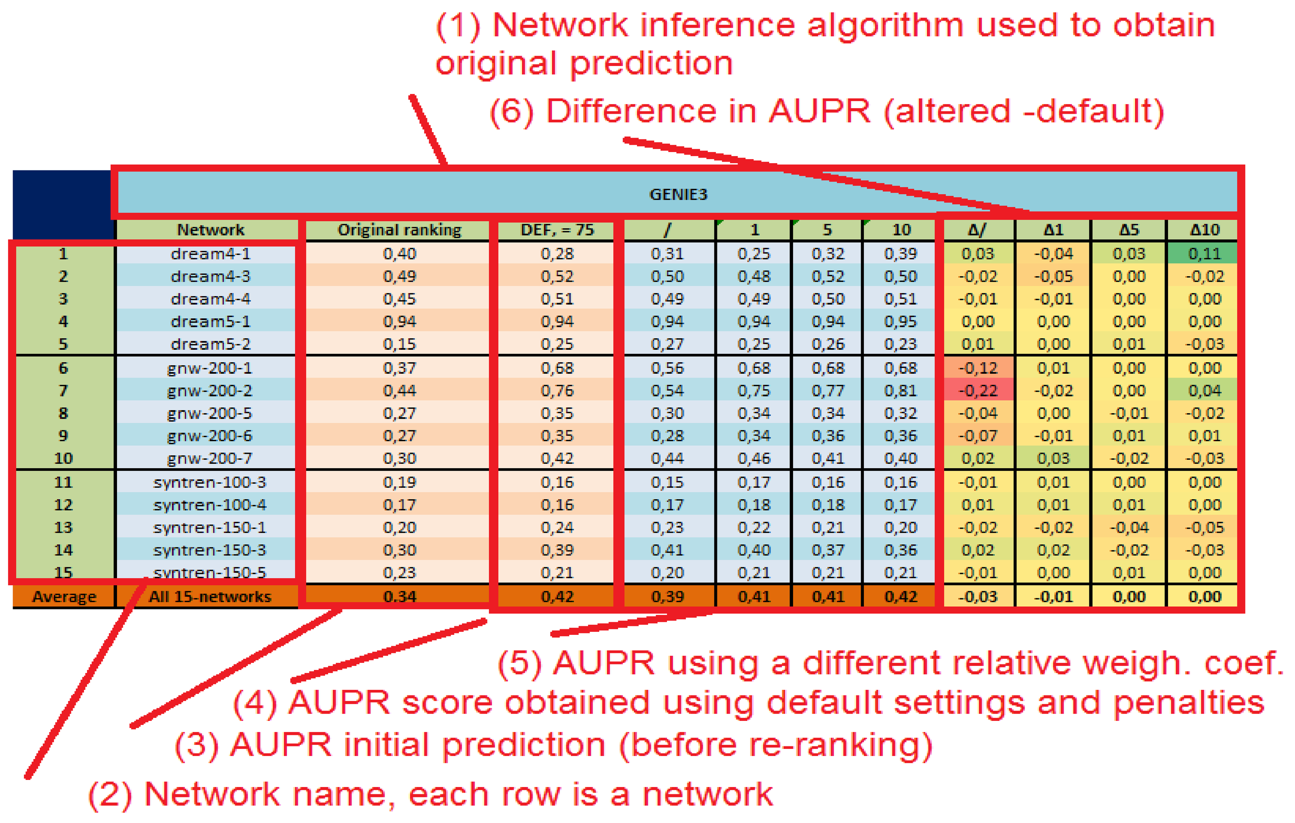


| | GENIE3 | | | | | | | | | | |
|---|---|---|---|---|---|---|---|---|---|---|---|
| | Network | Original ranking | DEF, = 75 | / | 1 | 5 | 10 | Δ/ | Δ1 | Δ5 | Δ10 |
| 1 | dream4-1 | 0,40 | 0,28 | 0,31 | 0,25 | 0,32 | 0,39 | 0,03 | -0,04 | 0,03 | 0,11 |
| 2 | dream4-3 | 0,49 | 0,52 | 0,50 | 0,48 | 0,52 | 0,50 | -0,02 | -0,05 | 0,00 | -0,02 |
| 3 | dream4-4 | 0,45 | 0,51 | 0,49 | 0,49 | 0,50 | 0,51 | -0,01 | -0,01 | 0,00 | 0,00 |
| 4 | dream5-1 | 0,94 | 0,94 | 0,94 | 0,94 | 0,94 | 0,95 | 0,00 | 0,00 | 0,00 | 0,00 |
| 5 | dream5-2 | 0,15 | 0,25 | 0,27 | 0,25 | 0,26 | 0,23 | 0,01 | 0,00 | 0,01 | -0,03 |
| 6 | gnw-200-1 | 0,37 | 0,68 | 0,56 | 0,68 | 0,68 | 0,68 | -0,12 | 0,01 | 0,00 | 0,00 |
| 7 | gnw-200-2 | 0,44 | 0,76 | 0,54 | 0,75 | 0,77 | 0,81 | -0,22 | -0,02 | 0,00 | 0,04 |
| 8 | gnw-200-5 | 0,27 | 0,35 | 0,30 | 0,34 | 0,34 | 0,32 | -0,04 | 0,00 | -0,01 | -0,02 |
| 9 | gnw-200-6 | 0,27 | 0,35 | 0,28 | 0,34 | 0,36 | 0,36 | -0,07 | -0,01 | 0,01 | 0,01 |
| 10 | gnw-200-7 | 0,30 | 0,42 | 0,44 | 0,46 | 0,41 | 0,40 | 0,02 | 0,03 | -0,02 | -0,03 |
| 11 | syntren-100-3 | 0,19 | 0,16 | 0,15 | 0,17 | 0,16 | 0,16 | -0,01 | 0,01 | 0,00 | 0,00 |
| 12 | syntren-100-4 | 0,17 | 0,16 | 0,17 | 0,18 | 0,18 | 0,17 | 0,01 | 0,01 | 0,01 | 0,00 |
| 13 | syntren-150-1 | 0,20 | 0,24 | 0,23 | 0,22 | 0,21 | 0,20 | -0,02 | -0,02 | -0,04 | -0,05 |
| 14 | syntren-150-3 | 0,30 | 0,39 | 0,41 | 0,40 | 0,37 | 0,36 | 0,02 | 0,02 | -0,02 | -0,03 |
| 15 | syntren-150-5 | 0,23 | 0,21 | 0,20 | 0,21 | 0,21 | 0,21 | -0,01 | 0,00 | 0,01 | 0,00 |
| Average | All 15-networks | 0,34 | 0,42 | 0,39 | 0,41 | 0,41 | 0,42 | -0,03 | -0,01 | 0,00 | 0,00 |
