# Supplementary material for: Netter: re-ranking gene network inference predictions using structural network properties
Source: BMC Bioinformatics. 2016 Feb 9;17:76. doi: 10.1186/s12859-016-0913-0 (PMC4746913; doi:10.1186/s12859-016-0913-0)
Supplement: Additional file 2 — Add 2 Regulatory Penalty.pdf. Additional file 2 is a pdf file containing the default mapping of the regulatory gene penalty and a detailed view of the stability tests. (PDF 411 KB) [file 12859_2016_913_MOESM2_ESM.pdf]

Regulatory gene cost function shape variability analysis

We investigate how the definition of the cost function influences the performance on a randomly reduced subset of 15 networks. The penalty mapping is replaced by 16 different simple penalty mappings as shown below. All other parameters and penalty mappings were set to the default values. The AUPR scores are shown and compared to the scores obtained by using the default settings.

Default penalty function definition

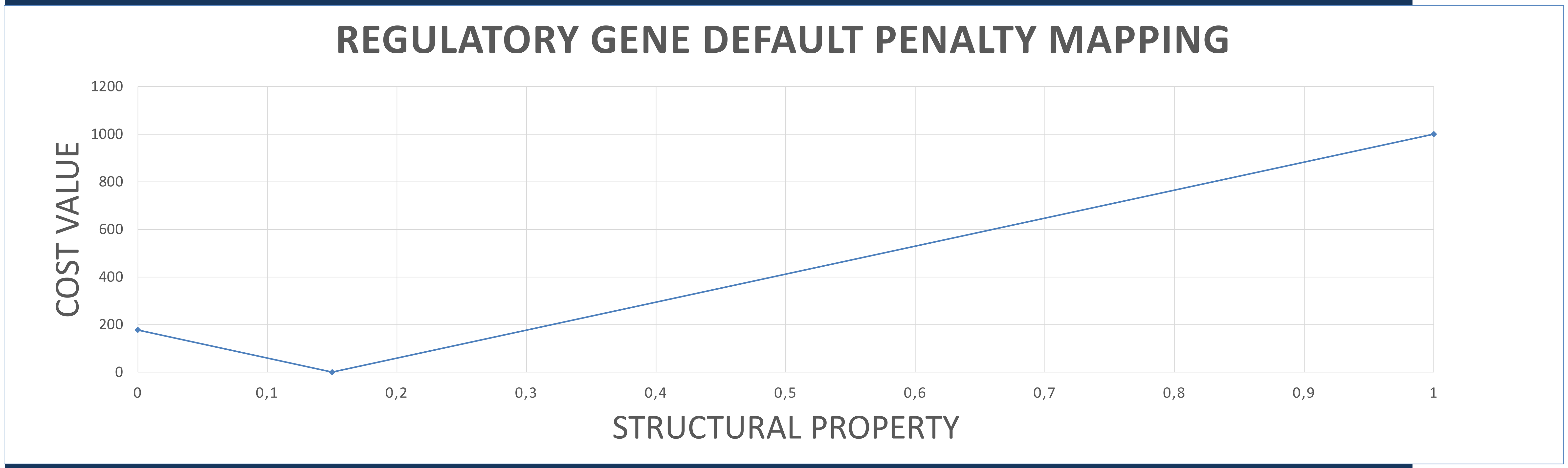

Stability testing - Visualization of the grid search functions

We visualize the other penalty mappings which were used in the 4 by 4 grid search in the stability tests.

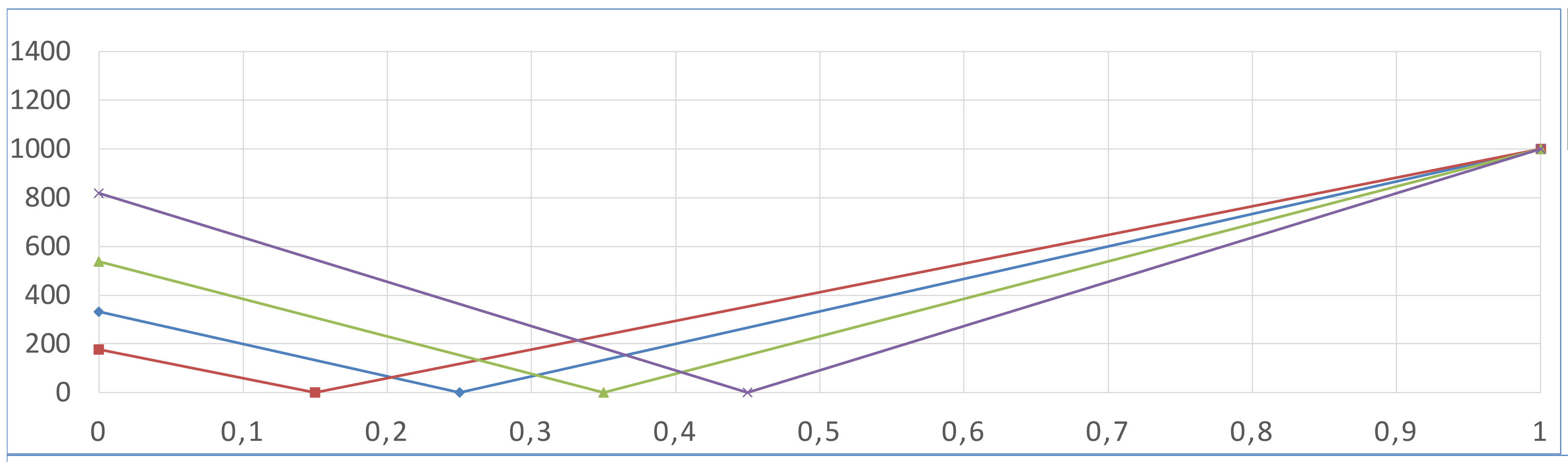

1		2 (DEFAULT)		3		4	
Key Value	Cost	Key Value	Cost	Key Value	Cost	Key Value	Cost
0	333,333	0	176,471	0	538,462	0	818,182
0,25	0	0,15	0	0,35	0	0,45	0
1	1000	1	1000	1	1000	1	1000

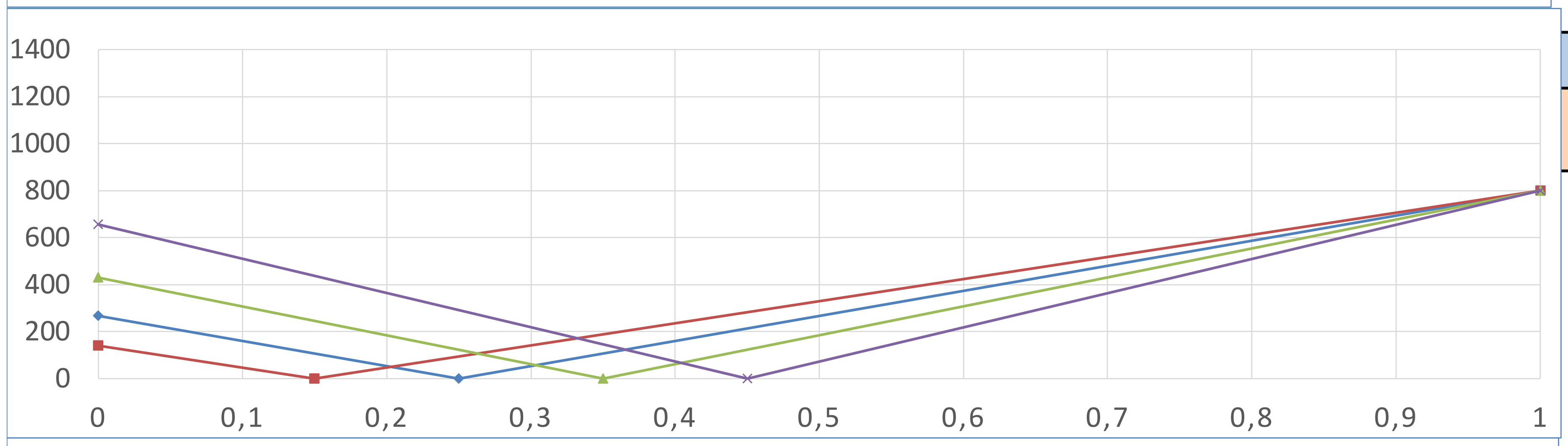

5		6		7		8	
Key Value	Cost	Key Value	Cost	Key Value	Cost	Key Value	Cost
0	266,667	0	141,176	0	430,769	0	654,545
0,25	0	0,15	0	0,35	0	0,45	0
1	800	1	800	1	800	1	800

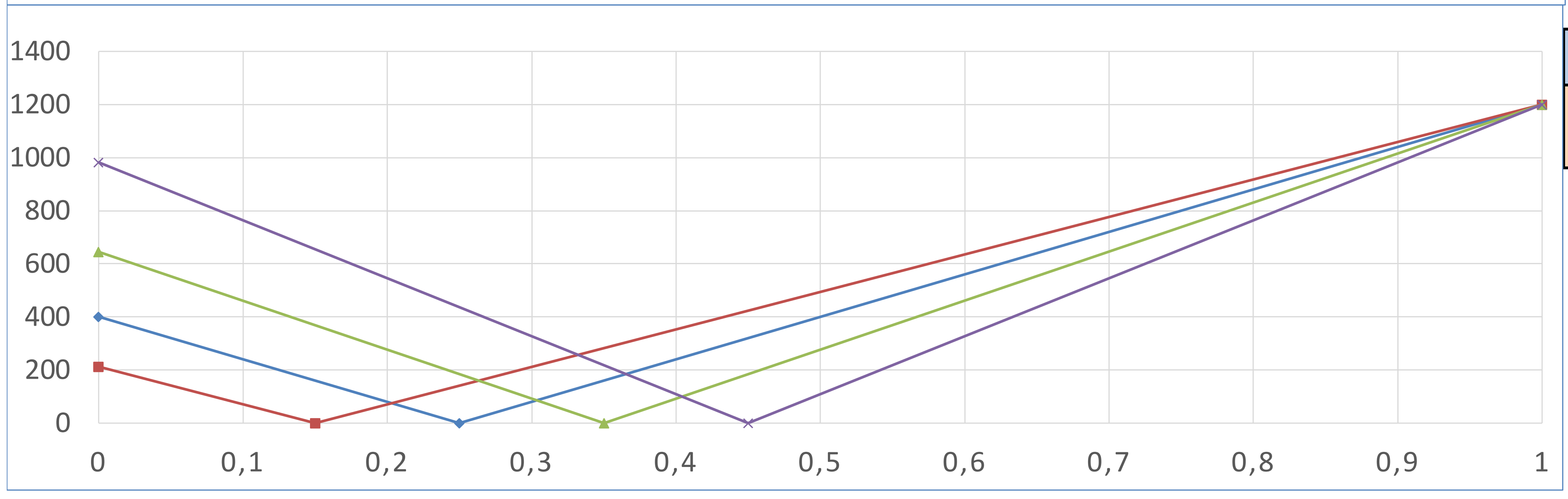

9		10		11		12	
Key Value	Cost	Key Value	Cost	Key Value	Cost	Key Value	Cost
0	400	0	211,765	0	646,154	0	981,818
0,25	0	0,15	0	0,35	0	0,45	0
1	1200	1	1200	1	1200	1	1200

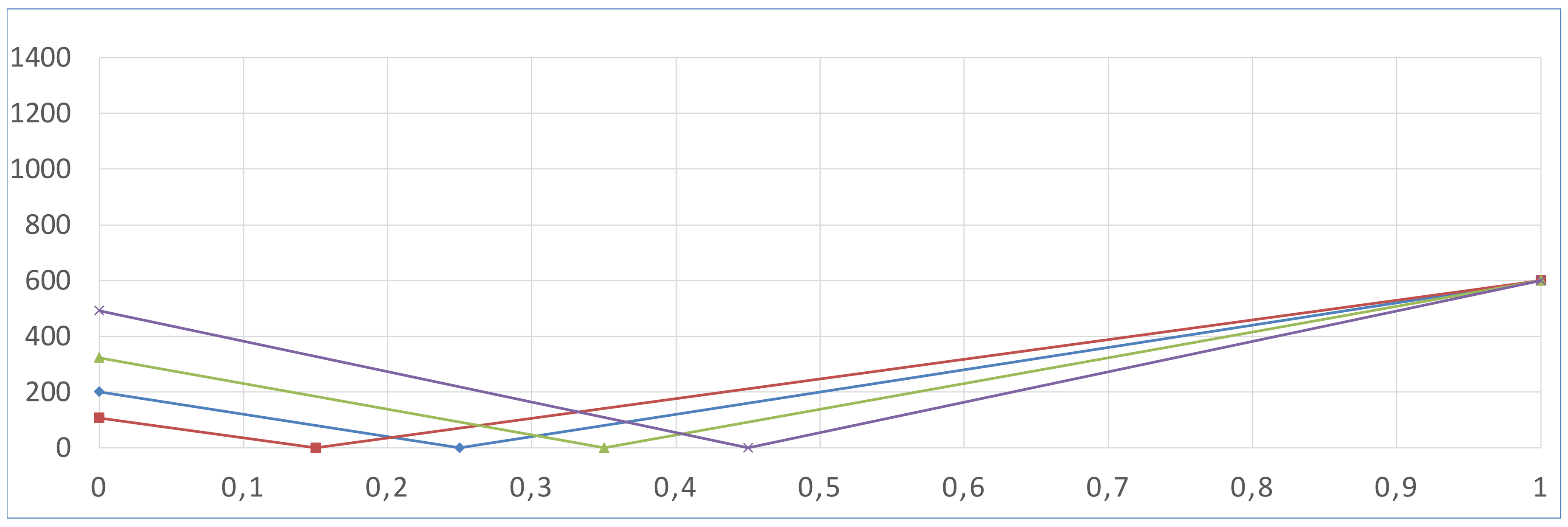

13		14		15		16	
Key Value	Cost	Key Value	Cost	Key Value	Cost	Key Value	Cost
0	200	0	105,882	0	323,077	0	490,909
0,25	0	0,15	0	0,35	0	0,45	0
1	600	1	600	1	600	1	600

Stability testing - Indivudual re-ranking AUPR scores

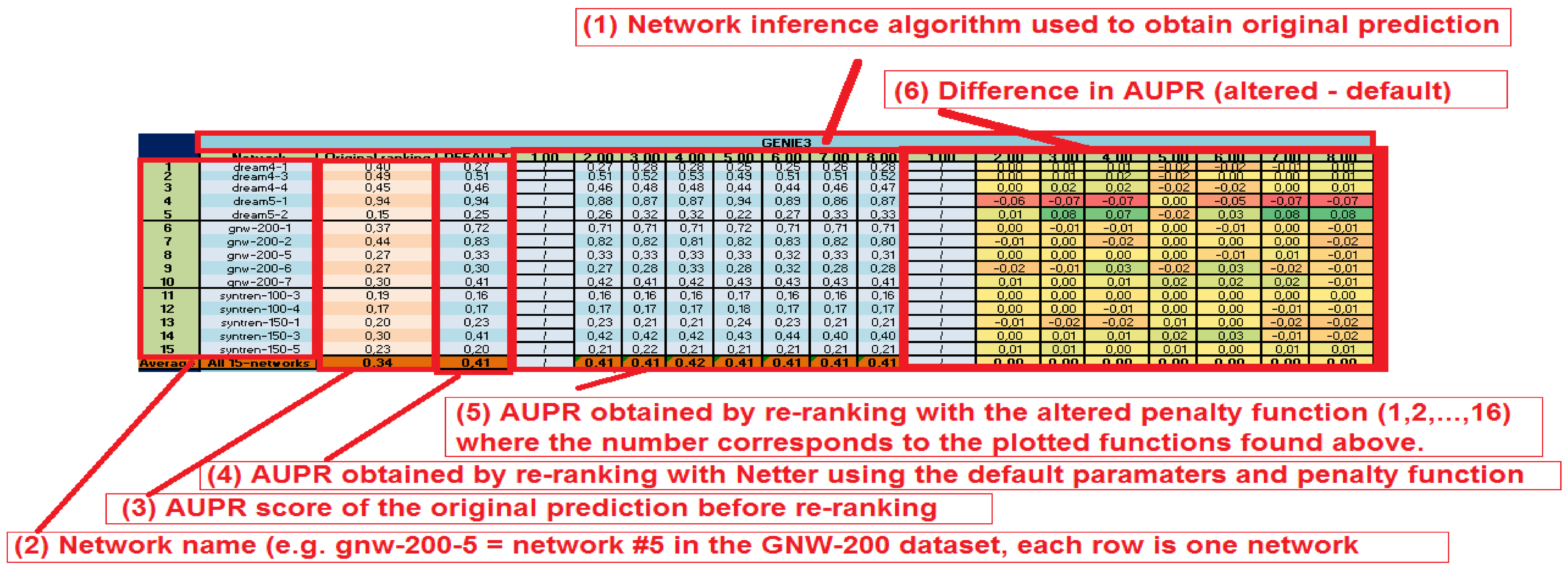

The table below lists the AUPR evaluation of the original prediction, the re-ranked prediction using default settings and all re-rankings obtained by using one of the altered penalty mappings found above. Each row is a network, each column is a re-ranking using a different setting.

	GENIE3																		
	Network	Original ranking	DEF, = 0,00001	1,00	2,00	3,00	4,00	5,00	6,00	7,00	8,00	1,00	2,00	3,00	4,00	5,00	6,00	7,00	8,00
1,00	dream4-1	0,40	0,27	0,30	/	0,27	0,29	0,30	0,29	0,29	0,29	0,03	/	0,00	0,02	0,03	0,02	0,02	0,02
2,00	dream4-3	0,49	0,51	0,50	/	0,51	0,51	0,52	0,54	0,51	0,52	-0,01	/	0,00	0,00	0,01	0,03	0,00	0,02
3,00	dream4-4	0,45	0,46	0,48	/	0,47	0,49	0,47	0,48	0,48	0,49	0,02	/	0,01	0,02	0,01	0,02	0,02	0,03
4,00	dream5-1	0,94	0,94	0,95	/	0,89	0,83	0,95	0,97	0,89	0,81	0,01	/	-0,05	-0,11	0,01	0,03	-0,05	-0,13
5,00	dream5-2	0,15	0,25	0,21	/	0,23	0,25	0,22	0,26	0,23	0,24	-0,03	/	-0,01	0,00	-0,03	0,01	-0,01	-0,01
6,00	gnw-200-1	0,37	0,72	0,71	/	0,71	0,70	0,72	0,71	0,71	0,69	0,00	/	-0,01	-0,02	0,01	-0,01	-0,01	-0,03
7,00	gnw-200-2	0,44	0,83	0,80	/	0,79	0,82	0,82	0,83	0,80	0,83	-0,03	/	-0,03	0,00	-0,01	0,00	-0,02	0,00
8,00	gnw-200-5	0,27	0,33	0,32	/	0,33	0,34	0,33	0,33	0,34	0,33	-0,01	/	0,00	0,01	0,01	0,00	0,01	0,00
9,00	gnw-200-6	0,27	0,30	0,30	/	0,33	0,31	0,27	0,27	0,32	0,29	0,00	/	0,04	0,02	-0,03	-0,03	0,02	-0,01
10,00	gnw-200-7	0,30	0,41	0,43	/	0,45	0,44	0,43	0,43	0,40	0,44	0,02	/	0,04	0,03	0,02	0,02	-0,02	0,02
11,00	syntren-100-3	0,19	0,16	0,16	/	0,16	0,16	0,16	0,16	0,16	0,16	0,00	/	0,00	0,00	0,00	0,00	0,00	0,00
12,00	syntren-100-4	0,17	0,17	0,17	/	0,17	0,17	0,17	0,16	0,17	0,17	0,00	/	0,00	-0,01	-0,01	-0,01	0,00	0,00
13,00	syntren-150-1	0,20	0,23	0,21	/	0,22	0,22	0,21	0,21	0,22	0,22	-0,02	/	-0,01	-0,01	-0,02	-0,02	-0,01	-0,01
14,00	syntren-150-3	0,30	0,41	0,42	/	0,42	0,42	0,43	0,41	0,42	0,41	0,00	/	0,00	0,01	0,02	-0,01	0,01	0,00
15,00	syntren-150-5	0,23	0,20	0,21	/	0,21	0,21	0,21	0,21	0,21	0,23	0,00	/	0,00	0,00	0,00	0,01	0,00	0,02
Average	All 15-networks	0,34	0,41	0,41	/	0,41	0,41	0,41	0,42	0,41	0,41	0,00	/	0,00	0,00	0,00	0,00	0,00	0,00

	GENIE3																		
	Network	Original ranking	DEF, = 0,00001	9,00	10,00	11,00	12,00	13,00	14,00	15,00	16,00	9,00	10,00	11,00	12,00	13,00	14,00	15,00	16,00
1,00	dream4-1	0,40	0,27	0,29	0,31	0,27	0,27	0,30	0,34	0,32	0,31	0,02	0,04	0,00	0,00	0,03	0,07	0,05	0,04
2,00	dream4-3	0,49	0,51	0,50	0,51	0,50	0,52	0,51	0,51	0,52	0,51	-0,01	0,00	-0,01	0,01	0,00	0,00	0,01	0,00
3,00	dream4-4	0,45	0,46	0,48	0,48	0,48	0,49	0,46	0,47	0,48	0,47	0,01	0,02	0,02	0,02	0,00	0,01	0,02	0,01
4,00	dream5-1	0,94	0,94	0,95	0,97	0,89	0,83	0,95	0,97	0,89	0,81	0,01	0,03	-0,05	-0,11	0,01	0,03	-0,05	-0,13
5,00	dream5-2	0,15	0,25	0,20	0,25	0,25	0,24	0,23	0,24	0,23	0,23	-0,05	0,00	0,00	-0,01	-0,02	-0,01	-0,02	-0,01
6,00	gnw-200-1	0,37	0,72	0,71	0,70	0,70	0,71	0,72	0,74	0,72	0,70	-0,01	-0,02	-0,01	-0,01	0,00	0,02	0,00	-0,02
7,00	gnw-200-2	0,44	0,83	0,81	0,81	0,80	0,82	0,84	0,84	0,83	0,84	-0,02	-0,02	-0,02	-0,01	0,01	0,01	0,00	0,02
8,00	gnw-200-5	0,27	0,33	0,33	0,32	0,34	0,34	0,33	0,29	0,33	0,32	0,00	0,00	0,01	0,01	0,00	-0,04	0,00	-0,01
9,00	gnw-200-6	0,27	0,30	0,30	0,33	0,31	0,29	0,27	0,26	0,25	0,29	0,01	0,04	0,01	-0,01	-0,03	-0,04	-0,04	-0,01
10,00	gnw-200-7	0,30	0,41	0,42	0,40	0,43	0,45	0,42	0,41	0,44	0,40	0,00	-0,01	0,02	0,04	0,00	0,00	0,03	-0,01
11,00	syntren-100-3	0,19	0,16	0,16	0,16	0,16	0,16	0,16	0,16	0,16	0,16	0,00	0,00	0,00	0,00	0,00	0,00	0,00	0,00
12,00	syntren-100-4	0,17	0,17	0,17	0,17	0,17	0,17	0,17	0,17	0,17	0,17	0,00	0,00	-0,01	-0,01	-0,01	-0,01	-0,01	-0,01
13,00	syntren-150-1	0,20	0,23	0,22	0,22	0,22	0,22	0,21	0,23	0,21	0,22	-0,01	-0,01	-0,01	-0,01	-0,02	0,00	-0,02	-0,02
14,00	syntren-150-3	0,30	0,41	0,42	0,41	0,42	0,42	0,40	0,43	0,42	0,41	0,00	0,00	0,01	0,01	-0,01	0,01	0,00	0,00
15,00	syntren-150-5	0,23	0,20	0,21	0,21	0,21	0,21	0,21	0,22	0,20	0,21	0,00	0,00	0,01	0,00	0,01	0,01	0,00	0,00
Average	All 15-networks	0,34	0,41	0,41	0,42	0,41	0,41	0,41	0,42	0,41	0,40	0,00	0,00	0,00	0,00	0,00	0,00	0,00	-0,01

Stability testing - Varying the relative weighing parameter

We investigate what the effect is of making the penalty more or less important in the total cost function. This is done by varying the coefficient on a randomly reduced subset of 15 networks. AUPR values are shown.

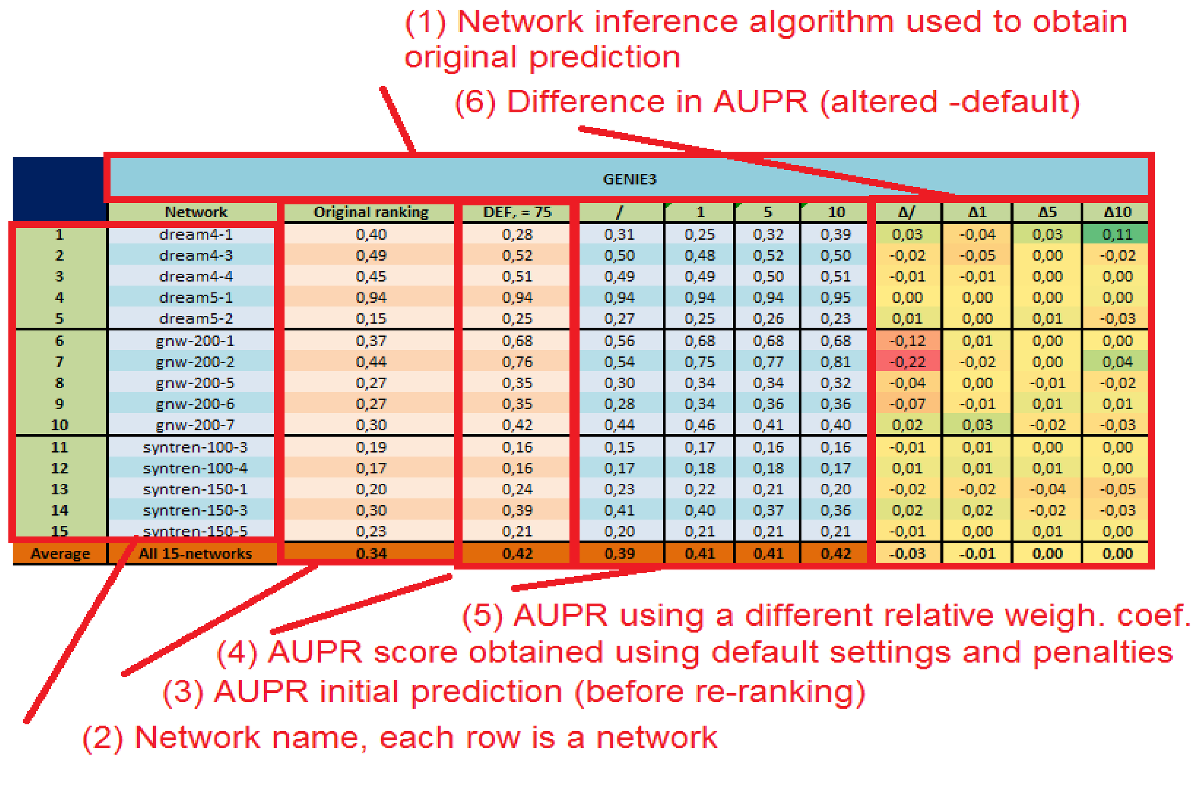

	GENIE3										
	Network	Original ranking	DEF, = 25	/	1	15	35	Δ/	Δ1	Δ15	Δ35
1	dream4-1	0,40	0,28	0,36	0,35	0,33	0,26	0,08	0,07	0,05	-0,02
2	dream4-3	0,49	0,52	0,46	0,43	0,51	0,49	-0,06	-0,09	-0,01	-0,03
3	dream4-4	0,45	0,51	0,44	0,45	0,48	0,48	-0,07	-0,05	-0,03	-0,03
4	dream5-1	0,94	0,94	0,93	0,94	0,95	0,95	-0,01	0,00	0,01	0,00
5	dream5-2	0,15	0,25	0,23	0,23	0,23	0,21	-0,03	-0,02	-0,02	-0,04
6	gnw-200-1	0,37	0,68	0,57	0,63	0,71	0,72	-0,11	-0,05	0,03	0,04
7	gnw-200-2	0,44	0,76	0,64	0,70	0,83	0,80	-0,13	-0,06	0,07	0,04
8	gnw-200-5	0,27	0,35	0,22	0,23	0,32	0,34	-0,13	-0,11	-0,03	-0,01
9	gnw-200-6	0,27	0,35	0,17	0,19	0,26	0,31	-0,18	-0,17	-0,09	-0,04
10	gnw-200-7	0,30	0,42	0,28	0,32	0,40	0,44	-0,15	-0,10	-0,02	0,02
11	syntren-100-3	0,19	0,16	0,15	0,15	0,16	0,16	-0,01	-0,01	0,00	0,00
12	syntren-100-4	0,17	0,16	0,17	0,17	0,16	0,17	0,00	0,00	0,00	0,01
13	syntren-150-1	0,20	0,24	0,20	0,20	0,20	0,22	-0,04	-0,04	-0,04	-0,02
14	syntren-150-3	0,30	0,39	0,35	0,39	0,41	0,41	-0,04	0,00	0,02	0,03
15	syntren-150-5	0,23	0,21	0,21	0,24	0,21	0,21	0,00	0,03	0,00	0,00
Average	All 15-networks	0,34	0,42	0,36	0,38	0,41	0,41	-0,06	-0,04	0,00	0,00
